# Tetrahedrite Nanocomposites for High Performance Thermoelectrics

**DOI:** 10.3390/nano15050351

**Published:** 2025-02-24

**Authors:** Rodrigo Coelho, Duarte Moço, Ana I. de Sá, Paulo P. da Luz, Filipe Neves, Maria de Fátima Cerqueira, Elsa B. Lopes, Francisco P. Brito, Panagiotis Mangelis, Theodora Kyratsi, António P. Gonçalves

**Affiliations:** 1 Centro de Ciências e Tecnologias Nucleares (C2TN), Departamento de Engenharia e Ciências Nucleares (DECN), Instituto Superior Técnico, Universidade de Lisboa, Campus Tecnológico e Nuclear, 2695-066 Bobadela, Portugal; rodrigo.coelho@ctn.tecnico.ulisboa.pt (R.C.); duarte_moco@sapo.pt (D.M.); eblopes@ctn.tecnico.ulisboa.pt (E.B.L.); 2 Laboratório Nacional de Energia e Geologia, I.P., Campus do Lumiar, Estrada do Paço do Lumiar, 22, 1649-038 Lisboa, Portugal; ana.sa@lneg.pt (A.I.d.S.); paulo.luz@lneg.pt (P.P.d.L.); filipe.neves@lneg.pt (F.N.); 3 International Iberian Nanotechnology Laboratory, 4715-330 Braga, Portugal; fatima.cerqueira@inl.int; 4 Centro de Física das Universidades do Minho e Porto (CF-UM-UP), Universidade do Minho, 4710-057 Braga, Portugal; 5 Mechanical Engineering and Resource Sustainability Center (MEtRICs), Departamento de Engenharia Mecânica (DEM), Universidade do Minho, 4800-058 Guimarães, Portugal; francisco@dem.uminho.pt; 6 Department of Mechanical and Manufacturing Engineering, University of Cyprus, 1678 Nicosia, Cyprus; mangelis.panagiotis@ucy.ac.cy (P.M.); kyratsi.theodora@ucy.ac.cy (T.K.)

**Keywords:** tetrahedrite nanocomposites, MoS_2_ nanoparticles, hot-pressing, lattice thermal conductivity, transport properties, weighted mobility, thermoelectric materials

## Abstract

Thermoelectric (TE) materials offer a promising solution to reduce green gas emissions, decrease energy consumption, and improve energy management due to their ability to directly convert heat into electricity and vice versa. Despite their potential, integrating new TE materials into bulk TE devices remains a challenge. To change this paradigm, the preparation of highly efficient tetrahedrite nanocomposites is proposed. Tetrahedrites were first prepared by solid state reaction, followed by the addition of MoS_2_ nanoparticles (NPs) and hot-pressing at 848 K with 56 MPa for a duration of 90 min to obtain nanocomposites. The materials were characterized by XRD, SEM-EDS, and Raman spectroscopy to evaluate the composites’ matrix and NP distribution. To complement the results, lattice thermal conductivity and the weighted mobility were evaluated. The NPs’ addition to the tetrahedrites resulted in an increase of 36% of the maximum figure of merit (zT) comparatively with the base material. This increase is explained by the reduction of the material’s lattice thermal conductivity while maintaining its mobility. Such results highlight the potential of nanocomposites to contribute to the development of a new generation of TE devices based on more affordable and efficient materials.

## 1. Introduction

The European Union is strongly committed to speeding up the energy transition to decarbonized systems, with the search for new environmentally friendly energy sources and the optimization of energy consumption being fundamental goals. Moreover, in the age of the Internet of Things (IoT) and Industry 4.0, countless sensors are a daily reality, and the development of devices without battery replacement or energy consumption from the grid is now a priority [1,2,3].

The discovery of new molecules and materials can be facilitators to achieve this new, more sustainable, world. In that regard, thermoelectric (TE) materials can directly convert thermal energy into electricity and, reversibly, electricity into thermal energy without the need for complex machinery. They can be used in many applications, one of the most evident being energy generation from heat, including waste heat and concentrated solar energy [4,5]. Moreover, the complete absence of moving parts, their noiselessness, and the absence of detrimental substances such as fluorinated cooling agents makes TE devices highly attractive [6]. Unfortunately, current commercial TE devices use materials developed in the 1960s, mainly based on Bi_2_Te_3_. However, their principal elements are rare and/or toxic, and their efficiency is quite low (<10%) [7,8]. Therefore, it is critical to identify new systems made of abundant, affordable, and environmentally friendly elements that could lead to higher device efficiencies.

The potential of a material for thermoelectricity can be evaluated by its dimensionless figure of merit, $zT=S^{2}\sigma T/\kappa$, where S represents the Seebeck coefficient, σ represents the electrical conductivity, and κ represents the total thermal conductivity. This last variable can be split into two contributions, $\kappa =\kappa_{L}+\kappa_{e}$, with $\kappa_{e}$ coming from electrons (electronic contribution) and $\kappa_{L}$ coming from phonons (lattice vibrations) [9]. Higher zTs would lead to higher device efficiencies, meaning that it is necessary to maximize the zT by increasing the power factor ($PF=S^{2}\sigma$) and/or decreasing κ. The PF depends on the carrier concentration (n) that passes through a maximum for highly degenerated small gap semiconductors and semimetals (n~10^19^ cm^−3^) [10,11]. For a given value of PF, the only way to increase the zT is by decreasing $\kappa_{L}$, since $\kappa_{e}$ is proportional to σ through the Wiedemann–Franz relationship [12].

The Bi_2_Te_3_-based commercial phases have zT values near 1 [13,14]. In the 90s, a second generation of TE materials was developed, with zT values of up to 1.7 [15,16,17]. A third generation of TE materials is now under investigation, combining different cutting-edge approaches to further increase the materials’ TE performance. These strategies include the increase of the PF through band engineering [18,19], optimizing grain boundary recrystallization processes [20], reducing $\kappa_{L}$ by combining nanostructures and microstructures based on hierarchical architectures [21,22], and employing decoupling carrier-phonon scattering techniques along with other advanced methods [23,24]. The maximum zT reported for bulk species is 2.6 [25], but unfortunately, they are unstable under working conditions.

In addition to a high zT, for their practical use, it is vital to consider the cost and ecological backpack of the elements and materials, since they can be prohibitive [26]. Unfortunately, both the commercial grade and most of the second and third generation of TE materials are expensive and often contain rare and/or toxic elements (i.e., Tl, Te, and Pb). This means that using current commercial TE devices for energy harvesting requires a considerable investment, with return on that investment being achieved only after long time periods (typically 4–8 years) [27,28,29]. Therefore, due to high costs, long recovery periods, and relatively low performance, the large-scale applicability of TE devices remains limited. Despite interest from various industrial sectors [30,31,32], commercial TE devices continue to be economically uncompetitive compared to electricity generation from solar and other green energy production systems.

Tetrahedrites are complex sulfosalts with Cu_12−x_(Zn,Fe,Ni,Mn,Co)_x_Sb_4_S_13_ general composition that are based on affordable, abundant, and low-toxicity elements [33,34,35,36,37,38]. They have high positive Seebeck coefficients (being *p*-type semiconductors), and big PFs can be obtained by adjusting their composition [34]. They crystallize in a cubic structure (space group I4̅3m, No. 217) with 58 atoms per unit cell, forming a sulfidic sodalite-like framework with Laves truncated tetrahedral holes [39,40]. This complex crystallographic structure leads to low κ [37,38], resulting in zT > 1 and placing the tetrahedrites among the bulk materials with the highest TE performances in the 300–650 K temperature range [37,38,41,42]. Their cost is low (~6 €/kg) when compared with the other most common materials, such as Bi_2_Te_3_ (>58 €/kg) [41], making them attractive for the development of new TE devices that can be used in large scale. Given the economic and environmental advantages of using tetrahedrite materials for energy harvesting, recent studies have explored the potential of natural tetrahedrite minerals for TE applications [43,44,45]. However, purifying these raw materials for obtaining good TE performances and meeting the requirements for device fabrication remains a challenge. Nevertheless, some research efforts have focused on combining selected natural and synthetic tetrahedrites, resulting in promising thermoelectric performances [44].

While band engineering has been widely used to increase PF through doping (or substitution), the intrinsically low κ of tetrahedrites leads to much lower interest in decreasing it even more by nanostructuring or using hierarchical architectures. Atomic-scale lattice disorder is inherent to solid solutions, and a decrease in $\kappa_{L}$ is observed for substitutions up to ~50% [46,47,48,49]. However, only a few studies on tetrahedrite-based nanocomposites have been reported so far [50,51,52], and no investigations on the decrease of $\kappa_{L}$ at mesoscales have been described.

The performance of oxide nanoparticles (NPs) dispersed on Cu_11.5_Ni_0.5_Sb_4_S_13-δ_ TE composites has been investigated, and zTs ~40–50% higher than the undoped tetrahedrite were achieved due to the interaction of phonons at the nanoscale [50,51,52]. This improvement is attributed to the addition of NPs, which reduce $\kappa_{L}$. However, a decrease in σ was also seen, though it was compensated by an increase in S. The decrease in σ can be explained by the large average electronegativity difference between the oxides and tetrahedrite, which reduces the charge carrier mobility [53]. Therefore, it is expected that the use of NPs with a lower average electronegativity difference would have a lower reduction effect and, consequently, lead to higher σ, while maintaining the decrease in $\kappa_{L}$.

In fact, such an effect was prominently demonstrated in the work of Bo Yang and his team [54], where various NPs, including SiC, diamond (C), B_4_C, and SnO_2_, were dispersed in Cu_11.5_Ni_0.5_Sb_4_S_12.7_ tetrahedrites. The addition of small amounts of non-oxide NPs led to a reduction in both κ and $\kappa_{L}$, with minimal impact in σ being observed. Although a slight reduction in σ still existed, it remained negligible for the composites containing SiC, diamond, and B_4_C NPs. In contrast, composites filled with oxide NPs, such as SnO_2_, exhibited a substantial σ reduction of approximately 38% near 300 K. These findings highlight the potential of further optimizing the tetrahedrite materials by using non-oxide NPs and nanostructuring strategies.

Following a similar line of investigation as the studies mentioned above, this work explores the hypothesis of enhancing the TE performance of Cu_11_Mn_1_Sb_4_S_13_ tetrahedrites by preparing composites with the addition of very stable nanosulfides, such as MoS_2_. The objective of the work is to explore an all-hierarchy approach [21,55], where the tetrahedrites with structures from nano to micro dimensions (pores, crystals, and grains) combined with the NPs can act as scattering centers to effectively reduce the lattice thermal conductivity and improve the materials zT. This strategy is expected to result in a significant improvement in TE performance, bringing the tetrahedrites closer to integration in a new and more affordable generation of commercial TE devices.

## 2. Materials and Methods

The tetrahedrite base materials were synthetized from small pieces of pure Cu (99.9999%, Alfa Aesar, Haverhill, MA, USA), Sb (99.9999%, Alfa Aesar, Haverhill, MA, USA), Mn (99.9%, Alfa Aesar, Haverhill, MA, USA), and S (99.5%, Alfa Aesar, Haverhill, MA, USA), which were weighed and mixed in stoichiometric ratios to prepare the nominal composition of Cu_11_Mn_1_Sb_4_S_13_. The mixtures were organized in several batches of 6 g each and sealed inside evacuated (10^−3^ Pa) quartz ampoules that were later moved to vertical furnaces. The ampoules were heated up to 1191 K at a rate of 6.5 K/min to melt the mixtures. The temperature was maintained for 1 h, with the ampoules being removed every 20 min, quickly agitated for homogenization of the melt, and placed back into the furnace. After 1 h, they were removed and left to cool down to room temperature. The obtained ingots were then manually crushed using an agata mortar and a pestle to obtain fine tetrahedrite powders.

The Cu_11_Mn_1_Sb_4_S_13_ composites were prepared inside a glovebox filled with argon gas by manually mixing the tetrahedrite powders with the MoS_2_ NPs (90 nm average particle size, 99% purity, 5.6 g/cm^3^, Sigma Aldrich, St. Louis, MO, USA). The weight ratios of NPs/tetrahedrite powders were 0, 0.1, 0.2, 0.3, 0.5, 0.8, and 1 wt%. The homogenization of the powders was performed using an agate mortar and pestle, and the resultant mixtures were placed in stainless steel molds (12 mm Ø, tungsten coated) that were cold-pressed (CP) at 512 MPa. The resultant CP pellets were sealed inside quartz tubes (10^−3^ Pa) and annealed in vertical furnaces at 713 K for 5 days.

At the end of the annealing process, the treated pellets were ground again to powders that were placed in graphite molds (10 mm Ø). The Cu_11_Mn_1_Sb_4_S_13_ composites were hot-pressed (HP) at 848 K with a pressure of 56 MPa for 90 min. To prevent the pellets from sticking to the graphite molds during sintering, two graphite sheets (130 µm thick, 99.8% purity, from Alfa Aesar) were placed between the powders and the graphite punches. After the HP step, dense pellets ($\rho_{RD}$ ≥ 90%) with 10 mm diameter and 3 mm thickness were obtained.

The pellets were polished using SiC sandpapers grits P600, P1000, P1200, P2500, and P4000 and cleaned with ethanol 70% vol before being characterized.

To identify the phase composition of the tetrahedrite composites, X-ray diffraction (XRD) analysis was performed. The measurements were carried out in a *Bruker D2 PHASEER* diffractometer (from Billerica, MA, USA) using a Bragg–Brentano geometry and Cu Kα radiation source (wavelength of 1.54060 nm). The surface of the pellets was analyzed by placing them on a sample holder that was levelled to mimic a powder measurement. The XRD experiment started by setting the current and tension to 30 kV and 10 mA, respectively. All data were collected on a 2θ range from 10° to 65° with a step of 0.02° and an acquisition time of 1.70 s per step. To decrease the influence of preferential orientation, the sample holder was rotated at a rate of 3 revolutions per minute. The acquired diffractograms were treated using *OriginPro* software version 9.0, and phase identification was performed by comparison of the observed data against cards from the *Crystallographic Open Database* (COD) using *DIFRAC.EVA* software (version 5.1).

Scanning electron microscopy analysis was carried out on *Phenom ProX G6 Desktop SEM* equipped with an EDS system (both from *Thermo Fisher Scientific*, Waltham, MA, USA). The surfaces of the pellets were observed using an accelerated voltage of 15 kV at a pressure of 1 Pa. Several micrographs of the materials were acquired in SE and BSE modes. To evaluate the chemical composition of the samples, EDS semi-quantitative analysis was carried out with an accelerating voltage of 15 kV. We estimate a relative error of 12% in the EDS analysis, which is within the acceptable limits for standardless analysis carried out on polished and slightly inclined surfaces [56]. To examine the porosity of the composites, three micrographs of the pellet surfaces were captured. These micrographs were acquired from the top, center, and bottom to ensure better reliability on the porosity calculation. The collected micrographs were treated using *ImageJ* software (version 1.5a), and the porosity was calculated using a particle analysis method [57,58].

Raman spectroscopy analysis was carried out to evaluate the NPs’ dispersion and interaction with the tetrahedrite matrix. Punctual analysis was performed with a *Labram HR 800 Evolution* confocal Raman microscope (from HORIBA France SAS, Palaiseau, France) equipped with an external 532 nm diode laser of 10 mW and a Peltier cooled CCD detector. All Raman spectra were acquired using grating of 600 groove/mm and by focusing the laser beam on the surface of the pellets with the help of a 100× objective lens. The laser power was set to ~1 mW, and the spot size is estimated to range between 1–2 μm. Most of the spectra were recorded in the range of 80–1700 cm^−1^, with an integration time of 10 s and 4 accumulations per spectrum. The MoS_2_ NPs powders were analyzed by dispersing them in microscope slides. The NPs spectra were acquired using an integration time of 10 s and 16 accumulations per spectrum.

The Raman maps of the composites were acquired using a *WiTec Alpha300 R* confocal Raman microscope (from WITec GmbH, Ulm, Germany) equipped with three excitation lasers (532 nm, 633 nm, and 785 nm) and two gratings (600 groove/mm and 1800 groove/mm). The surface of the pellets was scanned with the 532 nm Nd.YAG laser with a power in the range of 0.5–1.5 mW. The laser beam (1–2 μm spot size) was focused on the surface of the pellets using a 50× objective lens (from Zeiss, Jena, Germany). All the spectra were collected with a grating of 1800 groove/mm. The samples were first characterized in several sample points using 10 acquisitions with a 2 s acquisition time for the inspection of sample homogeneity. The Raman maps were generated by using the *Spectral Imaging Mode* of the *Alpha300 R*, where various spectra are acquired at each pixel by performing an XY scan with a range of between 50 × 50 μm and 50 × 50 pixels using a 50× objective lens with a 0.7 numerical aperture. For the acquisition of the maps, an integration time of 1s per spectrum was used. All the Raman maps presented in this work were obtained from the generated multi-spectrum file by using *WITec Project Plus* software (version 5.1).

The measurements of the transport properties (S, σ, and κ) were carried out on two pieces of equipment. The composites filled with 0, 0.3, 0.5, 0.8, and 1 wt% of NPs were measured on the *ULVACO-RIKO ZEM-3* system (from ULVAC-RIKO, Inc., Kanagawa, Japan), while the composites filled with 0.1 and 0.2 wt% were measured on the *Linseis LZT Meter* (combined LSR + LFA) system (from Linseis Messgeräte GmbH, Selb, Germany). The temperature dependence of the Seebeck coefficient (S) and electrical conductivity (σ) were recorded from room temperature to ~650 K. The thermal diffusivity of the samples was determined by the laser flash technique (following the ASTM E1461-01 standard [59,60]) under the same temperature range. For the diffusivity measurements, the *Netzsch LFA 457* equipment (from NETZSCH-Gerätebau GmbH, Selb, Germany) and the *Linseis LZT Meter* were used. The total thermal conductivity (κ) was estimated using the product between the thermal diffusivity, the density (measured by the Archimedes principle), and the heat capacity (Pyroceram 9606 comparison method and Dulong–Petit approximation). The lattice thermal conductivity ($\kappa_{L}$) was estimated by subtracting the electronic contribution from κ using the Wiedemann–Franz law ($\kappa =\kappa_{e}+\kappa_{L}=\kappa_{L}+L\sigma T$) and through the calculation of the Lorenz number ($L=1.5+exp(-\left|S\right|$/116)) [9,61]. The measurements’ uncertainties are estimated to be 7% for the Seebeck coefficient, 5% for electrical conductivity, 10% for thermal conductivity, and 12% for the figure of merit [62].

## 3. Results

### 3.1. Crystall Structure and Phase Composition

**Figure 1:**
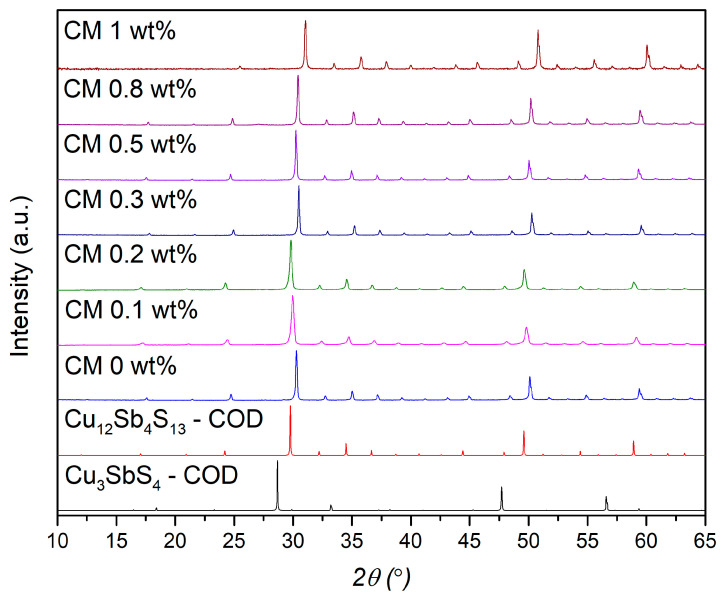
XRD diffractograms of the Cu_11_Mn_1_Sb_4_S_13_ composite pellets; tetrahedrite (# COD 8104303) and famatinite (# COD 8104122) pattern simulations are on the bottom.

The XRD diffractograms of the tetrahedrite composites (named “CM x wt%”, x = NPs amount) are displayed in Figure 1. According to the figure, all composites display a cubic tetrahedrite phase (space group I4̅3m, No. 217), as identified by COD card #8104303, which corresponds to a sulfidic sodalite-like (SOD) framework [39,40]. To complement the XRD analysis and confirm the phase purity of the composites, the pattern simulation of the famatinite phase (Cu_3_SbS_4_), which crystallizes into a tetragonal structure, space group I4̅2m (number 121), COD card # 8104122, is also presented. The XRD diffractogram of the MoS_2_ NPs is displayed in the Supplementary Data File, Figure S1, along with the MoS_2_ pattern simulation using the COD card # 9007660. According to COD database, the NPs have a hexagonal crystal structure and belong to the P 63/mmc space group (number 194). It can be seen that the MoS_2_ NPs are characterized by a high intensity peak appearing near 2θ = ~14°. The absence of peaks corresponding to NPs and other impurities in the composite diffractograms indicates that their detection was not possible, likely due to their low concentration and distribution, but the existence of such phases was confirmed by the SEM-EDS and Raman spectroscopy analysis, as shown below. It is important to mention that the XRD detection limit depends on several factors, such as phase composition, structure, fraction, crystallinity, and the analysis conditions. Additionally, since the XRD analysis was performed on pellets rather than powders, phase orientation may further hinder the detection of minor phases or nanoscale inclusions [63].

To evaluate the effect of NPs’ addition to the Cu_11_Mn_1_Sb_4_S_13_ tetrahedrite matrix, the cell parameters, crystallite size, and microstrain were analyzed. The cell parameters were calculated using the *UnitCell* program employing the method of T.J.B. Holland and S.A.T. Redfern [64]. The microstrain and average crystallite sizes were determined by employing the Williamson–Hall (W–H) method and the uniform deformation model (UDM) [65]. A comparative and qualitative analysis was made between all the composites. The W–H plots, crystallite size, and microstrain plots are provided in the Supplementary Data File, Figures S2 and S3. The resume of all data can be observed in Table 1.

From Table 1, it can be observed that the addition of MoS_2_ also leads to an increase in cell parameters, likely due to the creation of point defects such as dislocations, vacancies, or interstitials caused by the accommodation of NPs in the tetrahedrite matrix. Changes in the lattice parameters in thermoelectric composites are often attributed to the addition of NPs, with their interaction with the matrix leading to either an increase or a decrease in the lattice constant, as previously reported [66,67,68,69]. For example, in a recent study, Tian-Yu Yang and his team [66] reported that adding Fe_3_O_4_ particles to copper sulfides (Cu_1.8_S) results in the formation of vacancies, causing the crystal cell to expand and the lattice parameters to increase. This supports the hypothesis that defect formation in the tetrahedrite matrix is influenced by the quantity and dispersion of NPs.

Notably, the cell parameters displayed in Table 1 are also in the range of values for manganese-doped tetrahedrites reported in the literature (10.3700–10.4200 Å) [70,71,72,73,74]. This range arises from small variations in chemical composition due to doping at the 12d site. However, in the present work, the chemical composition of the tetrahedrite matrix is fixed. Therefore, small variations in stoichiometry (caused during synthesis) are not enough to explain the increasing trend observed for the mesh parameters. It is also important to note that to the best of our knowledge, there are no works reporting the existence of molybdenum-doped tetrahedrite materials. This fact excludes the possibility of variation of the lattice parameters due to the partial substitution of Cu by Mo into the tetrahedrite cell. Therefore, the possibility of reaction between the NPs and the tetrahedrites is very low, which was also one of the reasons why MoS_2_ NPs were chosen to prepare the composites.

Regarding the average crystallite size, in Table 1, it is possible to observe some variations with the addition of NPs. When compared with the base material (CM 0 wt%), most of the composites present reduced crystallite sizes. Interestingly, the smallest crystallite sizes are observed for CM 0.1 and CM 0.2 wt%. For higher additions of NPs, the average crystallite size increases, reaching the highest value for CM 0.5 wt%, and then decreases again for CM 0.8 and CM 1 wt%, as can be observed in Figure S2b from the Supplementary Materials Data File. The initial crystallite size reduction for lower NP concentrations agrees with other works reported in the literature for nanocomposite materials [51,75,76], suggesting that small amounts of NPs inhibit crystal growth by limiting the mobility of grain boundaries, giving origin to materials with a refined microstructure. Higher NP concentrations added to the tetrahedrite induces agglomeration (seen later in this work), which results in inhomogeneity and clustering of NPs. This effect allows crystallites to grow bigger during the sintering process since the pinning effect is reduced [77,78].

The lattice strain calculation (shown on the right-hand side of the table) displays results oscillating between positive and negative values without any visible trend. The reported oscillations can be related to the NPs’ dispersion and agglomeration, which change/increase the materials’ internal stresses and may promote the formation of localized point defects such as interstitials, vacancies, or dislocations that distort the tetrahedrite cell. This interpretation is consistent with the observed changes in cell parameters, suggesting that NPs’ addition and distribution significantly affect the material’s crystallographic structure. Moreover, according to some authors [65,79], the negative and positive strain values can indicate lattice deformation due to compressive strain or tensile strain. These different deformations indicate complex interactions between the tetrahedrite matrix and the NPs, which may result from dispersion and agglomeration effects.

**Figure 2:**
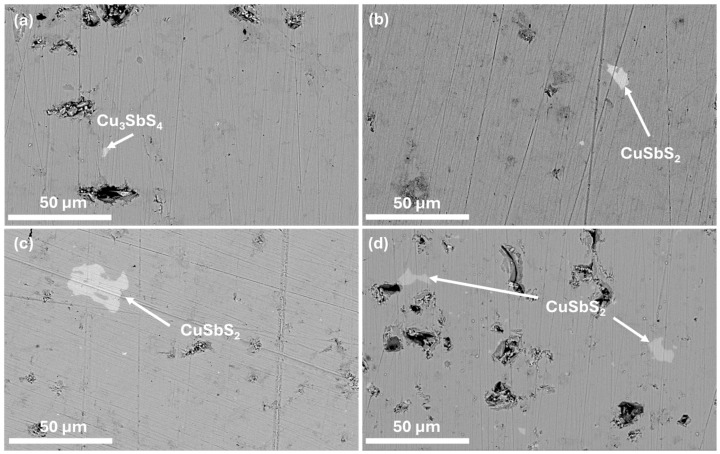
SEM-EDS micrographs of (**a**): CM 0 wt%; (**b**): CM 0.1; (**c**): CM 0.2; and (**d**): CM 0.3. Secondary phases are indicated by arrows. BSE mode 3000× magnification.

**Figure 3:**
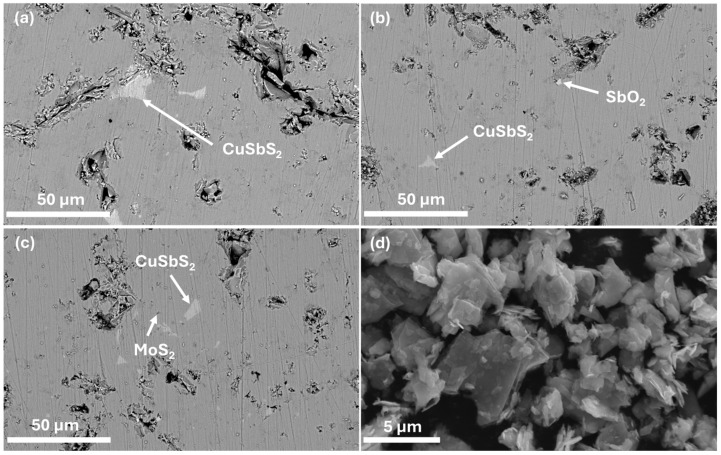
SEM-EDS micrographs of (**a**): CM 0.5 wt%; (**b**): CM 0.8; (**c**): CM 1; and (**d**): MoS_2_ nanopowder. Secondary phases are indicated by arrows. (**a**–**c**): BSE mode 3000× magnification and (**d**): 22,500× magnification.

To study the microstructure of the composites and complement the XRD analysis, SEM-EDS analysis was performed. The micrographs containing the polished surface of the composite pellets and of the MoS_2_ nanopowder are displayed in Figure 2 and Figure 3, where a homogeneous tetrahedrite phase with some porosity can be seen. Scattered across the tetrahedrites matrix, small quantities of secondary phases, such as SbO_2_ (antimony oxide) and CuSbS_2_ (chalcostibite), can be found. These phases are commonly associated with tetrahedrites [71,80] and typically appear within low amounts, explaining their absence in the XRD diffractograms. The chalcostibite phase results from small stoichiometry variations during synthesis, as can be deduced from the tetrahedrites’ ternary phase diagram [81,82]. The presence of oxygen (found in the form of an SbO_2_ secondary phase) can be explained by the small oxidation of the starting elements that can happen before synthesis.

Table 2 and Table 3 present the average EDS results and secondary phases detected in the composite materials. There are some small stoichiometry variations, but they lie within the analysis error. The specific EDS analysis of each composite material and MoS_2_ NPs is presented from Figures S4–S10 in the Supplementary Materials Data File.

The presence of MoS_2_ NPs was only spotted in CM 1 wt%, the composite with the highest NP content. The inability to identify the NPs’ phase in the other pellets was likely due to their low concentration, high dispersion, and/or similarity in contrast with the tetrahedrites matrix. The micrograph containing the MoS_2_ nanopowders is presented in Figure 3d. On this image, it is possible to observe that the NPs exhibit a flake-like morphology, presenting irregular and extremely thin sheets resembling graphene nanosheets. The thickness of the NPs appears to be only a few nanometers, while their lengths can range from a couple nanometers up to several micrometers, classifying the MoS_2_ NPs as 2D materials. On the same figure, it is also possible to notice some NP agglomerates of varied sizes reaching up to 6 µm in length. It is important to note that Figure 3d presents the SEM analysis of the MoS_2_ nanopowders dispersed in a carbon tape. Therefore, due to their size and high surface area, they tend to agglomerate.

Despite it not being possible to observe the NPs’ dispersion by SEM-EDS, their effect on the tetrahedrites matrix is evident when examining the composites’ microstructure (Figure 2 and Figure 3). In all pellets, a correlation between NPs’ addition and porosity can be found. For CM 0.1 and CM 0.2 wt%, porosity is minimal, whereas in the other composites, larger pores and debris become more prominent as the concentration of MoS_2_ NPs increases. The low porosity for CM 0.1 and CM 0.2 suggests the existence of an optimum NP concentration (and dispersion), where the crystallite growth is effectively suppressed without negatively affecting sintering. For higher NP amounts, the saturation limit within the tetrahedrite matrix is probably being reached, which then favors the agglomeration of NPs, leading to materials with poor mechanical quality due to increased porosity and cracks (that arise from the excess of NPs).

To more accurately characterize the porosity within the samples, several micrographs were analyzed using the *ImageJ* program and employing a particle analysis method. The micrographs used for porosity calculation can be observed in the Supplementary Data File from Figure S12–S18. The resume of the porosity analysis and the experimental density of the manganese composites (calculated by the Archimedes method) can be observed in Table 4.

According to Table 4, the porosity decreases for CM 0.1 and CM 0.2 and then increases from CM 0.3 to CM 1 wt%, confirming the hypothesis that the MoS_2_ NPs act as crystal inhibitors. In the middle column of Table 4, the green density of the pellets is presented. These values were calculated using a theoretical density of 4.933 g/cm^3^ for the tetrahedrite material [38]. As observed, all the pellets show values ≥ 95%. When examining the experimental density, fluctuations can be observed in the values, which is contrary to expectations. A possible explanation for this variation is the complex interaction between the tetrahedrite unit cell and the nanoparticles (NPs), leading to the introduction of point defects. This is also suggested by the XRD analysis, which shows changes in the cell parameters. At low NP concentrations, finer microstructures are formed, with pellets exhibiting smaller grain and crystallite sizes. The presence of point defects, combined with a high number of interfaces, may reduce the density due to atomic mismatches and less dense atomic packing [83,84]. At higher NP concentrations, dispersion is affected (as discussed below), leading to inhomogeneous distributions and NP aggregation/clustering. This results in coarser microstructures [77,78] and denser atomic packing due to fewer interfaces. Consequently, some composites exhibit experimental densities closer to the reference material (CM 0), creating the impression that lattice parameters, density, and MoS₂ content increase simultaneously. However, due to the complex microstructure of tetrahedrite composites after NP incorporation, it is not possible to establish a direct correlation between lattice parameters and density. Additionally, it is important to note that weight measurements for density determination were carried out in water, meaning that the liquid could partially or fully fill only the open pores, leading to an apparent increase in experimental density with increasing porosity. At the same time, the presence and effect of closed porosity in the materials cannot be ruled out.

**Figure 4:**
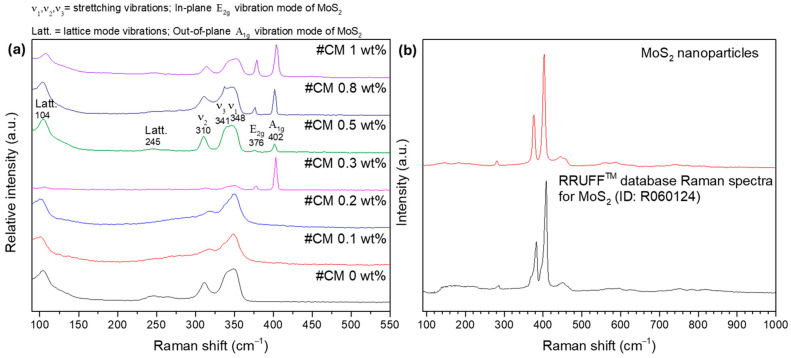
Room temperature Raman spectra of (**a**): composite materials (spot analysis) and (**b**): MoS_2_ nanoparticles.

4.797±0.0047.4±0.54.708±0.0020.9±0.34.683±0.0041.2±0.64.781±0.00310.7±0.64.758±0.00415.7±0.84.744±0.00219.3±1.04.765±0.00517.1±0.7 To study the NPs’ dispersion and complement the XRD and SEM-EDS results, Raman spectroscopy analysis was performed. In the first approach, the surface of the materials was investigated in multiple points to check samples’ homogeneity and look for the NPs’ signal. On a second approach, the surface of some pellets was investigated by scanning areas of 50 × 50 µm^2^ and generating the respective Raman maps. The resume of the spot analysis performed on the composites and the MoS_2_ nanopowder can be observed in Figure 4.

Most of the bands in the Raman spectra correspond to the tetrahedrite matrix Figure 4a, which exhibits a characteristic spectrum with convoluted peaks (or bands) ranging from 100 to 250 cm^−1^ and from 300 to 370 cm^−1^ [85,86,87,88]. The referred bands correspond to the lattice vibration modes and to the stretching and bending modes of the Sb-S and S-Sb-S bonds, respectively. According to the literature [85,86,89], the most intense bands in the tetrahedrites’ spectrum are typically broad and result from two or three overlapping (or convoluted) peaks, as illustrated on the spectrum of CM 0.5 wt%. In this material, a prominent band with a maximum around 348 cm^−1^ is observed. This maximum is attributed to the Sb-S symmetric stretching vibrations (named $\nu_{1}$) [86]. Another maximum near 341 cm^−1^ can be easily spotted, which is assigned to the antisymmetric stretching vibrations ($\nu_{3}$) [86]. Additionally, the band with the maximum near 310 cm^−1^ is associated with symmetric bending ($\nu_{2}$) of the S-Sb-S bonds [86]. Around 245 cm^−1^ and 104 cm^−1^, the bands associated with lattice mode vibrations can be observed [86].

Figure 4b shows the Raman spectrum of the MoS_2_ NPs acquired in a sample point. The NPs’ signal is clearly distinct from the composites matrix and can be typically identified by the presence of two sharp and intense bands appearing above 365 cm^−1^. These bands are assigned to the in-plane E_2g_ and out-of-plane A_1g_ vibration modes of the MoS_2_ NPs and agree with spectra for MoS_2_ materials available in *RRUFF* mineralogy database #ID R060124 [90].

While looking at all the tetrahedrite bands displayed in Figure 4a, it is possible to see that in some cases, the bands are slightly shifted, with peaks that can be overlapped and/or with different shapes. These small band shifts, overlaps, and shapes can result from small compositional changes between the composites and due to the complex interactions between the NPs and the tetrahedrites matrix. At the same time, a correlation between the NPs’ concentration and the intensity of the MoS_2_ bands can be seen. The higher the concentration of NPs in the composites, the more intense the MoS_2_ peaks. However, in the CM 0.3 wt% composite, the NP bands exhibit an unusually high intensity that goes out of trend. This anomaly is likely region-specific because if the laser targets a larger particle or an agglomerate, higher intensity is expected. On the samples CM 0.1 and CM 0.2, the presence of the MoS_2_ NPs was not found, possibly due to their low number and distribution.

Curiously, in CM 0.1 and CM 0.2, the peaks near 310 and 341 cm^−1^ seem to be slightly shifted to higher wavenumbers, indicating a blue shift. According to the literature [91,92,93,94,95], the Raman blue shift is typically associated with phonon confinement, compressive strain, and small crystallite sizes. These findings are also supported by the XRD and SEM-EDS results, further suggesting the formation of point defects in the tetrahedrites’ crystal cell that are likely enhanced by the addition of MoS_2_ NPs in low amounts.

**Figure 5:**
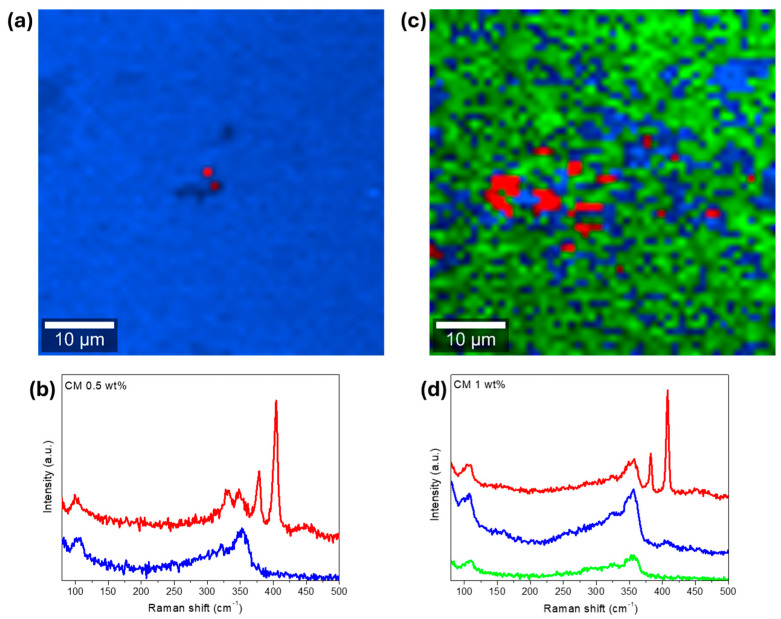
Raman analysis of composite CM 0.5 wt%, (**a**): Raman map (2 clusters of data), and (**b**): basis Raman spectra of the scanned area. On the right, CM 1 wt%, (**c**): Raman map (3 clusters of data), (**d**): basis Raman spectra of the scanned area. Laser power set to 0.5 mW and analyzed area of 50 µm × 50 µm. Each pixel color in the Raman maps corresponds to the color of the corresponding basis Raman spectra.

The Raman maps, whose pixel color corresponds to the color of the corresponding basis Raman spectra, are presented in Figure 5, for two composites. In the Raman map of CM 0.5 wt%, Figure 5a, only a small agglomerate (or particle) can be seen near the center of the micrograph. This particle seems to range between 1 and 2 µm, which agrees with the SEM-EDS observations. The absence of NPs on the other regions of the analyzed area help explain the large crystallite sizes evidenced by the XRD analysis. So, typically, MoS_2_ NPs inhibit crystal growth. However, when agglomeration occurs, the samples end up with poor NP dispersion, resulting in regions with unimpeded crystallite growth (or localized defect formations), which explains why the average crystallite sizes are higher on the pellets filed with high MoS_2_ NP content.

On the Raman map of CM 1 wt%, Figure 5c, larger MoS_2_ particles or agglomerates up to 6 µm in size can be seen. According to the Raman map, the NPs are not homogenously dispersed along the composite matrix, being concentrated in specific locations near the middle of the analyzed area/micrograph. At the same time, a weaker signature of the MoS_2_ NPs can be observed by the areas depicted in blue, as evidenced by the Raman spectra displayed in Figure 5d. These results show an improved NP distribution for CM 1 wt% compared with CM 0.5 wt% and explain the differences in the average crystallite size observed for the two samples.

In summary, based on all the analysis carried out so far, the addition of higher amounts of NPs to the tetrahedrite matrix typically results in agglomeration (saturation is achieved within 0.3 and 0.5 wt% of MoS_2_), bad NPs distribution, and increased porosity, leading to fragile materials, which also probably translates into poor TE performances.

### 3.2. Transport Properties

**Figure 6:**
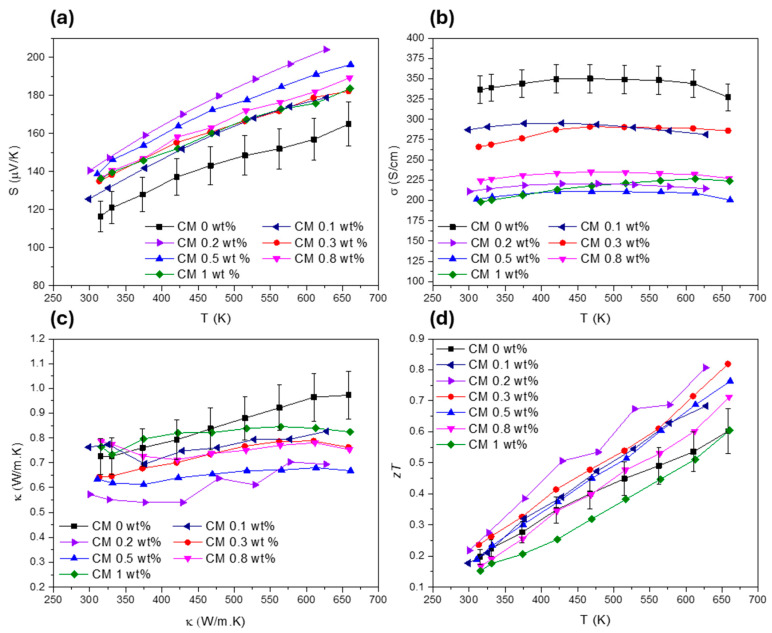
Transport properties of all the Cu_11_Mn_1_Sb_4_S_13_ composites, (**a**) Seebeck coefficient, (**b**) electrical conductivity, (**c**) total thermal conductivity, and (**d**) figure of merit.

To evaluate the TE performance of the composites, the transport properties were measured. The Seebeck coefficient (S), electrical conductivity (σ), total thermal conductivity (κ), and figure of merit (zT) for all the pellets are presented in Figure 6. Globally, S increases for all the composites with the addition of NPs. The composites with the highest Seebeck coefficient are CM 0.2 and CM 0.5 wt%, which present an increase of about 24% near room temperature. For the other composite materials, the thermopower also improved by up to 10% compared to the base tetrahedrite (the Cu_11_Mn_1_Sb_4_S_13_ composition—named CM 0 wt%) but oscillates for different amounts of MoS_2_. The observed variations of S can be attributed to the combination of several factors introduced by the addition of the MoS_2_ NPs. These factors are probably related to the microstructure refinement (since reduced crystallite sizes influence carrier concentration and mobility), which can promote energy filtering effects and boost phonon scattering [96,97]. Other factors include NP dispersion within the tetrahedrites matrix, which can affect the charge carrier scattering mechanisms and the composites band structure by shifting the valance and conduction bands or increasing the density of states near the Fermi level [98]. It should be noted that *S* is very sensitive to small changes in the band filling around the Fermi level, much more than the electrical conductivity.

Regarding σ, the highest values are attributed to CM 0.2 and CM 0.3, while the remaining composites present much lower values. In general, the electrical conductivity is reduced with the addition of NPs for all composites (by ~15% for CM 0.2 and CM 0.3 and by up to 40% for the rest of the materials). The increase of S and the decrease of σ are proportional, or of the same order, as the values observed for other tetrahedrite composites filled with B_4_C, SnO_2_, C (diamond), SiC, ZnO, and Nb_2_O_5_ [50,51,54]. The decrease in electrical conductivity for the CM composites can be explained by the reduced compaction (increase in porosity due to the addition of NPs), the increased number of interfaces within the materials, and the formation of point defects such as vacancies, as suggested by the microstructural analysis.

According to Figure 6c, the total thermal conductivity was successfully reduced in all the composites, with the lowest values being achieved for CM 0.2 and CM 0.5 wt%. Notably, the use of MoS_2_ nanosheets resulted in the suppression of the total thermal conductivity from 0.73 to near 0.56 (W/m.K) at room temperature. These results place the CM 0.2 and CM 0.5 composites among those with the lowest thermal conductivity reported in the literature for tetrahedrite composites as of the submission date, presenting values close to the ones reported for the Cu_11.5_Ni_0.5_Sb_4_S_13_ tetrahedrite filled with Fe_2_O_3_ NPs [52].

Due to reduced κ, improved S, and balanced σ, the composites with the highest zT were CM 0.2 and CM 0.3, which reached a maximum figure of merit of 0.81 near 625 K. This represents an increase of about 36% for the maximum zT when comparing with the base tetrahedrite (CM 0 wt%). Curiously, the highest zT values are achieved for the composites filled with the smallest concentration of NPs. These results agree well with the observations made in the previous section, which indicated that defects such as interstitials, vacancies, and dislocations should be potentiated for lower NP concentrations, suggesting a saturation limit with the tetrahedrite matrix for the addition of NPs.

## 4. Discussion

### 4.1. Lattice Thermal Conductivity Evaluation

**Figure 7:**
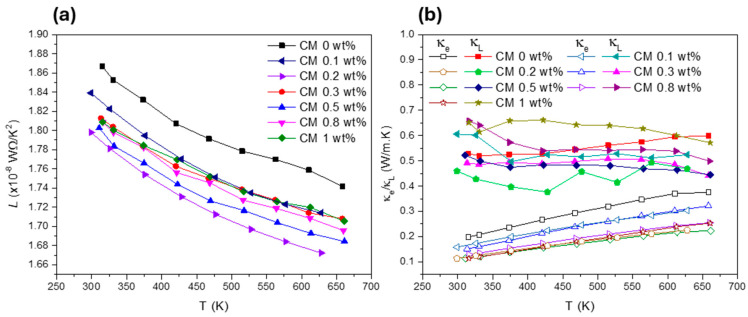
Lorenz number (**a**) and electronic and lattice thermal conductivities (**b**) for Cu_11_Mn_1_Sb_4_S_13_ composites.

To better understand how the NP concentration affects κ, the lattice thermal conductivity ($\kappa_{L}$) was calculated using the Wiedemann–Franz relationship. The graphs displaying the Lorenz number (L), as well as the lattice and electronic thermal conductivities ($\kappa_{L}$ and $\kappa_{e}$), are presented in Figure 7. Figure 7a shows that for all composite materials, L is comprehended below the degenerate limit for semiconductors (L < 2.44 × 10^−8^ WΩ/K^2^) [61]. At the same time, a reduction of $\kappa_{L}$ with the addition of NPs can be seen (Figure 7b). However, an exception to this tendency if observed for CM 1 wt% composite, which displays higher $\kappa_{L}$ values than the reference tetrahedrite (CM 0 wt%). The lowest $\kappa_{L}$ values are achieved for CM 0.2 wt%, which has a lattice thermal conductivity around 0.45 W/m·K in the entire temperature range. This behaviour is closely followed by CM 0.3 and CM 0.5 wt%, which are some of the materials with the highest zT.

Figure 7b shows that κ is dominated by $\kappa_{L}$, which means that the crystallographic structure and all the defects associated to the crystal cell play a significative role in the total thermal conductivity of the composites. The dependency of $\kappa_{L}$ on the defects can be explained using the Debye–Callaway model, which is represented by Equation (1) [99,100,101]:

(1) $$\kappa_{L}=\frac{k_{b}}{2\pi^{2}\nu }{\left(\frac{k_{b}}{h}\right)}^{3}\int_{0}^{\theta_{D}/T}\tau (x)\frac{e^{x}x^{4}}{{\left(e^{x}-1\right)}^{2}}d(x)$$

In the presented equation, $k_{b}$ represents the Bolztman constant, h represents the Plank constant, ν represents the sound velocity, $\theta_{D}$ represents the Debye temperature, and $\tau \left(x\right)$ represents the phonon relaxation time. By applying Matthiessen’s rule [9], it is possible to rewrite Equation (1) in function of the phonon relaxation time and transform it into the individual sum of all the contributing scattering mechanisms that affect $\kappa_{L}$, as exemplified by Equation (2). According to this equation, the total phonon relaxation time can depend on Umklapp scattering ($\tau_{U}^{-1}$), boundary scattering ($\tau_{B}^{-1}$), defects or impurity scattering ($\tau_{D}^{-1}$), normal scattering ($\tau_{N}^{-1}$), and mass fluctuation scattering $\tau_{MF}^{-1}$ [48].

(2) $${\tau (x)}^{-1}=\tau_{U}^{-1}+\tau_{B}^{-1}+\tau_{D}^{-1}+\tau_{N}^{-1}+\tau_{MF}^{-1}$$

In summary, Umklapp scattering (also called U-scattering) is an intrinsic phonon–phonon interaction that limits thermal conductivity. This scattering mechanism is characterized by phonon momentum loss due to collisions between phonons. The boundary scattering is related to the phonons that are scattered at the material’s boundaries. This scattering mechanism is particularly significant in nanostructures such as nanointerfaces (NPs, nanocrystals/grains) and the presence of different phases at the nanoscale. In this scattering mechanism, the phonon’s mean free path is proportional to the material’s dimensions, meaning that smaller grains or an increased number of interfaces enhance phonon scattering. Defects or impurity scattering occurs due to the existence of NPs, point defects, twin boundaries, staking faults, and other types of defects that can be present in the material’s matrix and crystal cell. These defects can alter phonon dynamics due to localized mass and strain variations, which disrupt the propagation of heat. Curiously, point defects can also affect electron scattering, which helps to explain the reduction of electrical conductivity observed in the Cu_11_Mn_1_Sb_4_S_13_ composites. The normal scattering (or N-scattering) is a type of phonon–phonon interaction where the phonon’s momentum is conserved and redistributed within the system. This scattering mechanism balances the phonon’s energy, attenuating heat conduction. Mass fluctuation scattering (MF scattering) occurs typically in doped materials where the substitutional atoms present higher masses than the original ones, which can disrupt heat transfer. A similar effect can also be promoted by defects that can also create mass fluctuations within the crystal cell. However, the efficacy in disrupting heat conduction can be localized in the case of defects, while substitutional atoms maintain their periodicity in the bulk and act at the atomic scale.

To better explain thermal transport in nanostructured materials, the different scattering mechanisms (associated to the phonon relaxation times presented in Equation (2)) can be further simplified by comparing them to their corresponding phonon angular frequencies (ω). For example, defects and nanostructures exhibit phonon scattering with a relaxation time $\tau_{D}^{-1}\propto \;\omega^{-1}$ and $\omega^{-3}$, respectively, which target mid-range frequency phonons [48,50,51,52,54]. Similarly, grain boundaries cause phonon scattering with relaxation times $\tau_{B}^{-1}\propto \;\omega^{0}$ targeting low-frequency phonons [48,50,51,52,54]. The tetrahedrites’ particular crystallographic structure, composed by Sb[CuS_3_]Sb trigonal bipyramids, contain Cu(2) rattlers that are effective in phonon scattering at lower frequencies, where the U- and N-scattering processes have relaxation times $\tau_{U,N}^{-1}\propto \;\omega^{-2}$ [48]. Additionally, the presence of dopants in the tetrahedrites cell, as well as other defects, like vacancies and interstitials, are capable of introducing mass-fluctuation scattering mechanisms that have relaxation times $\tau_{MF}^{-1}\propto \;\omega^{-4}$ particularly affecting scattering at high frequencies. Taking into consideration all these scattering mechanisms, multiple scattering processes can be present in the CM composites, which can explain the lattice thermal conductivity reduction and the improvement of the TE properties. However, the evaluation of charge carrier transport is also important due to the high porosity observed in the composites.

### 4.2. Mobility Evaluation and Overall Performance

To understand all the different scattering mechanisms (electron and phonon scattering), weighted mobility was calculated using a method proposed by G. Jeffrey Snyder and his team [102]. By using this method, it is possible to infer whether the increase in the composites’ zT is solely due to a reduction in thermal conductivity (caused by phonon scattering) or if other charge carrier scattering mechanisms are influencing the materials’ performance. Weighted mobility ($\mu_{w}$) is calculated directly from the TE’s properties using the Seebeck coefficient (S), resistivity (ρ), the Boltzmann constant ($k_{b}$), and the electron charge (e), as shown in Equation (3):

(3) $$\mu_{w}=331\frac{{cm}^{2}}{Vs}\left(\frac{mΩcm}{\rho }\right){\left(\frac{T}{300\;K}\right)}^{-\frac{3}{2}}\left[\frac{\exp\left[\frac{\left|S\right|}{k_{b}/e}-2\right]}{1+\exp\left[-5\left(\frac{\left|S\right|}{k_{b}/e}-1\right)\right]}+\frac{\frac{3}{\pi^{2}}\frac{\left|S\right|}{k_{b}/e}}{1+\exp\left[5\left(\frac{\left|S\right|}{k_{b}/e}-1\right)\right]}\right]$$

**Figure 8:**
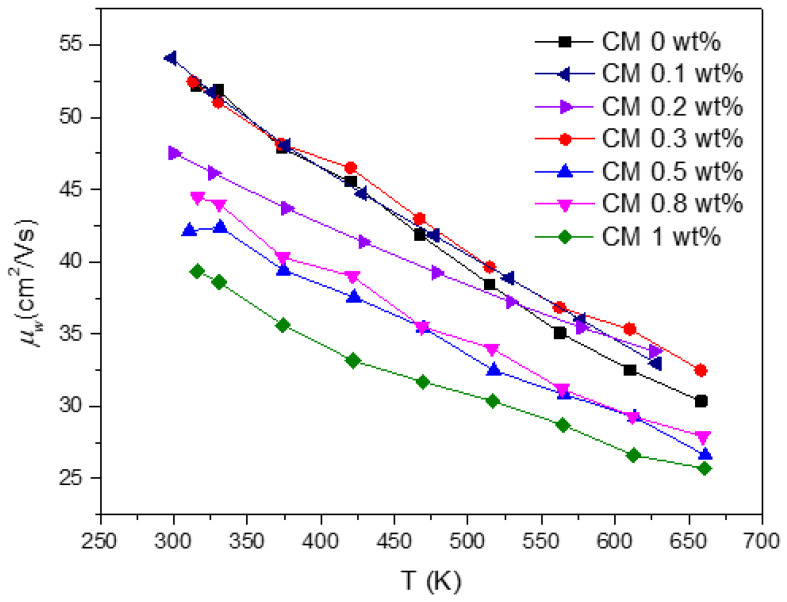
CM composites’ weighted mobility in function of temperature.

The weighted mobility for all the composites is presented in Figure 8. The data show that $\mu_{w}$ generally decreases with the addition of NPs. As previously discussed, the incorporation of MoS_2_ NPs introduces defects into the tetrahedrite’s matrix, results in smaller crystallite sizes, and increases porosity, which also affects the composites’ electrical conductivity. Pores are well known for their ability to effectively scatter phonons [72], contributing to a reduction in lattice thermal conductivity. However, increased porosity also reduces electrical conductivity as a side effect, as observed in the TE property measurements (displayed in Figure 6b). According to the weighted mobility calculations and to the analysis carried out so far, it can be inferred that the decrease in σ mostly affects the composites richer in MoS_2_ content and results from poor compaction and diminished grain contact, which together with the pores, enhances the scattering of charge carriers. Additionally, reduced crystallite sizes also further contribute to the electrical conductivity reduction. This occurs because an increased number of interfaces in TE materials typically leads to the reduction of the charge carrier’s mobility (increasing scattering). Therefore, it is possible to conclude that the addition of NPs introduces two competing scattering mechanisms: phonon scattering and electron scattering (or charge carrier scattering).

Surprisingly, the composites with low NP concentrations, such as CM 0.1, 0.2, and 0.3 wt%, exhibit the higher weighted mobility values (on par with the reference material). These findings are outstanding since, due to the elevated number of interfaces, especially for CM 0.1 and CM 0.2, the weighted mobility should be affected. In these particular cases, the good mobility values may result from an optimal quantity and dispersion of NPs, which should promote the creation of defects that improve charge carrier pathways or reduce the electron/hole scattering mechanisms. To evaluate this hypothesis, Raman maps were acquired on the surface of CM 0.2 and CM 0.3 wt%.

**Figure 9:**
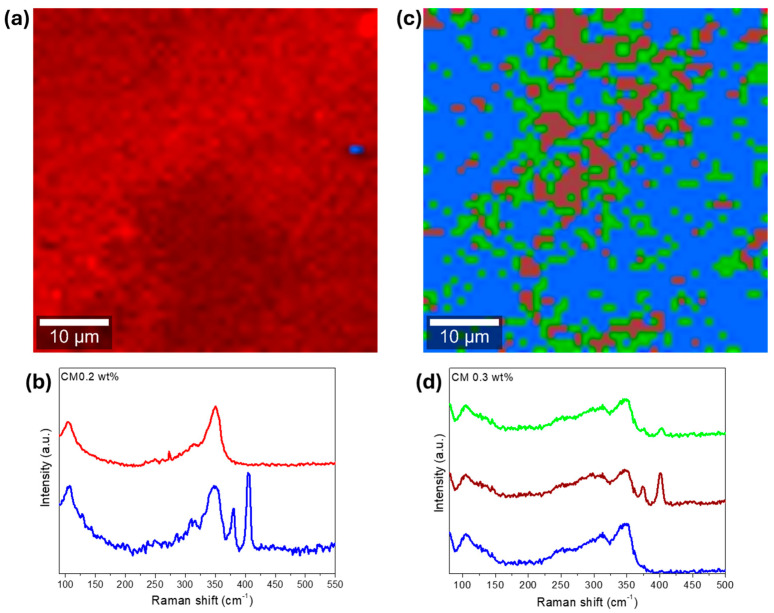
Raman analysis of composite CM 0.2 wt%, (**a**) Raman map (2 clusters of data), and (**b**) corresponding Raman basis spectra of the Raman map. On the right CM 1 wt%, (**c**) Raman map (3 clusters of data), (**d**) corresponding Raman basis spectra of the Raman map. Laser power set to 1 mW for CM 0.2 and 1.5 mW for CM 0.3 wt%. Each pixel color in the Raman maps corresponds to the color of the corresponding basis Raman spectra.

The Raman maps and corresponding Raman basic spectra for CM 0.2 and CM 0.3 are displayed at Figure 9. On the corresponding Raman basic spectra, a clear signal from both the tetrahedrite matrix and MoS_2_ NPs can be identified. However, during analysis, the laser interacted with the surface of the samples, causing some “burning” of the scanned area. This was resultant from the need to increase the laser power to be able to detect NPs on the composite materials filled with lower concentrations of MoS_2_.

On the Raman map for CM 0.2 (Figure 9a), a small agglomerate or particle that is 2 µm in size can be spotted. At first glance, the NPs appear almost absent (CM 0.2) and probably agglomerated (CM 0.3). However, in the Raman map constructed only with the NP Raman signature, provided in Figure S19 available in the Supplementary Data File, a good NP distribution can be observed for CM 0.2 wt%. This good distribution explains the reduced crystallite sizes and suggests the formation of defects, as already discussed. On the Raman map of CM 0.3 wt% (Figure 9d), several agglomerates up to 10 µm in size can be observed. Nevertheless, the MoS_2_ NPs seem to be distributed around all the tetrahedrite matrix, supporting the hypothesis of having better NP distributions for lower concentrations of MoS_2_, as predicted from the weighted mobility analysis. The high degree of MoS_2_ agglomeration found in Figure 9c probably results from the interaction between the laser and sample since all the scanned area was burned.

To form a general sense of the performance of the composite materials and to evaluate their potential for TE applications, the average zT was calculated using Equation (4). By integrating the zT over a narrow temperature range and considering the entire temperature measurement range as the materials’ operation temperature (effective ΔT), this approach provides a more realistic analysis of the composites’ TE performance under practical operating conditions.

(4) $${zT}_{avg}=\frac{\int_{T_{c}}^{T_{h}}zT\;(T)dT}{T_{h}-T_{c}}$$

**Figure 10:**
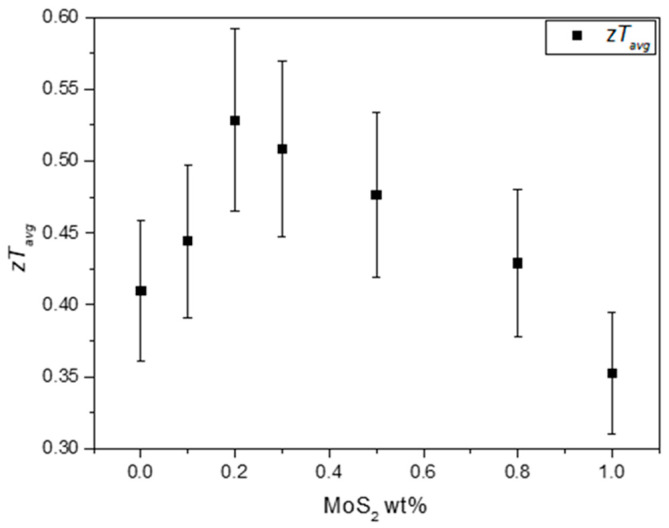
Average figure of merit for the Cu_11_Mn_1_Sb_4_S_13_ composites in function of the addition of MoS_2_ NPs.

The ${zT}_{avg}$ in function of the addition of NPs to the composite materials is displayed in Figure 10. With the addition of MoS_2_ NPs, the composites’ ${zT}_{avg}$ is enhanced from 0.41 up to 0.53, reaching the maximum value for CM 0.2 wt%. After reaching this maximum, the average figure of merit gradually decreases as higher amounts of NPs are incorporated into the tetrahedrites matrix. These results indicate that there is no advantage in preparing composites with higher contents of NPs, since the largest increase in performance are registered for low concentrations. Such results agree with several other reports on tetrahedrite composites [50,51,52,54]. In most of these studies, small concentrations of NPs typically lead to the higher performances. In this study, the observed increase for the ${zT}_{avg}$ accounts as an enhancement of ~29% in the overall TE performance compared to the Cu_11_Mn_1_Sb_4_S_13_ tetrahedrite material. If TE devices were manufactured, the device using the tetrahedrite nanocomposites would present a significant boost in energy conversion.

**Figure 11:**
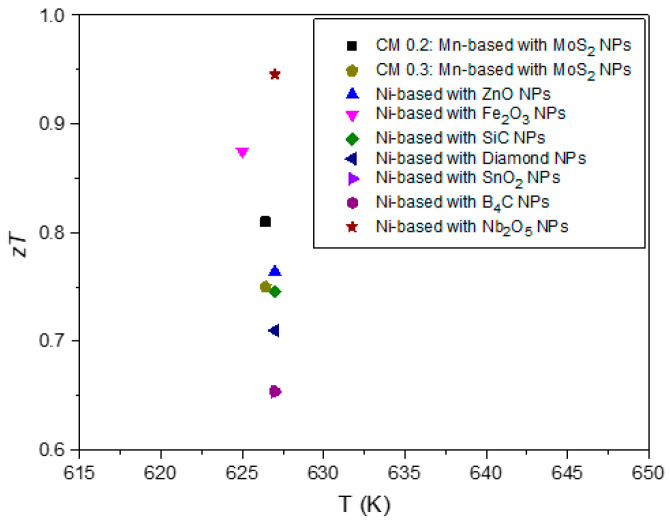
Scatter plot containing a comprehensive summary of the zT for all published studies on tetrahedrite composites to date [50,51,52,54]. Maximum zT analysed at 630 K.

To facilitate a comparison between the materials in this study and other tetrahedrite composites reported in the literature, Figure 11 presents a scatter plot summarizing the zT values from all published studies on tetrahedrite composites to date. The values were retrieved at a temperature of ~630 K to enable a direct comparison with manganese-doped tetrahedrite composites. As can be seen, the CM 0.2 and CM 0.3 composites are among the high-performance materials reported so far. Interestingly, the most used material in studies involving tetrahedrite composites is the nickel-doped tetrahedrite with the chemical composition of (Cu_11.5_Ni_0.5_Sb_4_S_13_), which is a tetrahedrite composition that typically displays zT values higher than the Cu_11_Mn_1_Sb_4_S_13_ tetrahedrite but that is less stable and, consequently, less suitable for applications [103].

## 5. Conclusions

Based on the XRD, SEM-EDS, and Raman spectroscopy analyses, it can be concluded that the addition of MoS_2_ NPs to the Cu_11_Mn_1_Sb_4_S_13_ tetrahedrite matrix induces the formation of defects, promotes the refinement of the materials’ microstructure, and increases porosity. According to the TE performance evaluation, the addition of NPs in low quantities (up to 0.3 wt%) increases the materials’ zT by reducing the lattice thermal conductivity, increasing the Seebeck and balancing the electrical conductivity. The dispersion and agglomeration of the MoS_2_ NPs (within the tetrahedrite matrix) play a significant role in the materials’ quality and performance. Generally, higher amounts of NPs result in higher agglomeration, low compaction, and poor TE performances, while small NP amounts result in better particle distributions, increased compaction, and higher TE performances.

When evaluating the Cu_11_Mn_1_Sb_4_S_13_ composite materials through an all-hierarchy approach, it is evident that materials present features capable of effectively suppressing the thermal conductivity from the atomic up to the mesoscale. For example, the presence of doping elements such as Mn and point defects (including vacancies, interstitials, and dislocations) can create mass and strain fluctuations capable of affecting phonon and charge carrier’s transport from the atomic up to the microscale. At the same time, the addition of NPs reduces crystallite sizes, increasing the number of interfaces within the materials and resulting in a microstructure refinement that intensifies the phonon scattering mechanisms without significantly affecting charge carrier transport. These multiple microstructures and nanostructures, including NPs, pores, interstitials, nanocrystals, multiple boundaries, and secondary phases, are likely to scatter phonons from low to high frequencies, resulting in an effective reduction of the lattice thermal conductivity that can be defined as $\tau \;\sim \;\omega^{0}+\omega^{-1}+\omega^{-3}+\omega^{-4}$ according to the Debye–Callaway model [9,48,99]. These scattering mechanisms (with a broad range of frequencies) can reduce the composites’ total thermal conductivity without severely affect the other transport properties, resulting in an enhanced TE performance, as supported by the weighted mobility calculations. Such evidence is also reinforced by observation of the so-called phonon confinement effect, which, according to Alexander Balandin and Kang L. Wang [94], can affect all phonon relaxation rates. To finish, it is important to mention that this work demonstrates the importance of exploring new types of NPs and different compositions of tetrahedrite materials. Following an all-hierarchy approach and through nanostructuring, it was possible to increase the average figure of merit up to 29% for the Cu_11_Mn_1_Sb_4_S_13_ tetrahedrites, contributing to the development of a new generation of TE materials that can be more affordable and easily implemented in energy harvesting for application in large scale.

## Figures and Tables

**Table 1:** Tetrahedrite composites’ cell parameters, average crystallite size, and microstrain.

Sample#	*a* (Å)	Average Crystallite Size—D (nm)	${ɛ}_{ms}\times 10^{-3}$
CM 0 wt%	10.4032±0.0007	75±6	0.03
CM 0.1 wt%	10.3900±0.0007	27±2	−0.36
CM 0.2 wt%	10.4058±0.0007	34±2	−0.75
CM 0.3 wt%	10.4164±0.0007	71±3	−0.01
CM 0.5 wt%	10.4115±0.0007	95±10	0.25
CM 0.8 wt%	10.4229±0.0007	70±5	0.02
CM 1 wt%	10.4336±0.0005	57±6	−0.26

**Table 2:** Average EDS results and detected secondary phases for the CM0, CM0.1, CM0.2, and CM0.3 composite pellets.

#Sample	Nominal Composition	Analyzed Composition	Detected Secondary Phases
**#CM 0**	Cu_11_Mn_1_Sb_4_S_13_	Cu_11.5±1.4_Mn_0.9±0.1_Sb_3.1±0.4_S_13.5±1.6_	Cu_2.2±0.3_Sb_1.5±0.2_S_4.2±0.5_
**#CM 0.1**	Cu_11_Mn_1_Sb_4_S_13_	Cu_10.4±1.3_Mn_1.1±0.1_Sb_2.9±0.3_S_14.6±1.8_	Cu_1.1±0.1_Sb_0.7±0.1_S_2.2±0.3_
**#CM 0.2**	Cu_11_Mn_1_Sb_4_S_13_	Cu_10.7±1.3_Mn_1.0±0.1_Sb_2.9±0.3_S_14.4±1.7_	Cu_1.1±0.1_Sb_0.7±0.1_S_2.2±0.3_
**#CM 0.3**	Cu_11_Mn_1_Sb_4_S_13_	Cu_11.7±1.4_Mn_0.9±0.1_Sb_2.7±0.3_S_13.7±1.6_	Cu_1.1±0.1_Sb_0.8±0.1_S_2.1±0.3_

**Table 3:** Average EDS results and detected secondary phases for the CM0.5, CM0.8, and CM1 composite pellets and NP powders.

#Sample	Nominal Composition	Analyzed Composition	Detected Secondary Phases
**#CM 0.5**	Cu_11_Mn_1_Sb_4_S_13_	Cu_11.7±1.4_Mn_1.0±0.1_Sb_3.0±0.4_S_13.3±1.6_	Cu_1.1±0.1_Sb_0.8±0.1_S_2.1±0.3_
**#CM 0.8**	Cu_11_Mn_1_Sb_4_S_13_	Cu_11.7±1.4_Mn_0.9±0.1_Sb_2.9±0.4_S_13.4±1.6_	Cu_1.2±0.1_Sb_0.7±0.1_S_2.1±0.2_Sb_0.9±0.1_O_1.8±0.2_
**#CM 1**	Cu_11_Mn_1_Sb_4_S_13_	Cu_11.2±1.3_Mn_1.0±0.1_Sb_2.8±0.3_S_14.0±1.7_	Cu_1.2±0.1_Sb_0.7±0.1_S_2.1±0.3_
**#NPs Powder**	MoS_2_	Mo_1.1±0.1_S_1.9±0.2_	-

**Table 4:** Tetrahedrite composites’ experimental density and SEM porosity.

#Sample	Experimental Density (g/cm^3^)	Green Density	SEM Porosity (%)
**CM 0 wt%**	4.797±0.004	97.24%	7.4±0.5
**CM 0.1 wt%**	4.708±0.002	95.44%	0.9±0.3
**CM 0.2 wt%**	4.683±0.004	94.93%	1.2±0.6
**CM 0.3 wt%**	4.781±0.003	96.92%	10.7±0.6
**CM 0.5 wt%**	4.758±0.004	96.45%	15.7±0.8
**CM 0.8 wt%**	4.744±0.002	96.17%	19.3±1.0
**CM 1 wt%**	4.765±0.005	96.59%	17.1±0.7

## Data Availability

Data is contained within the article and Supplementary Material.

## Supplementary Materials

The following supporting information can be downloaded at: https://www.mdpi.com/article/10.3390/nano15050351/s1, Figure S1: XRD analysis of the MoS_2_ NPs on the top and pattern simulation using COD card #9007660 on the bottom.; Figure S2: Tetrahedrite composites cell parameters (a), average crystallite size (b), and lattice strain (c).; Figure S3: W–H plots of the tetrahedrite composites employing the UDM.; Figure S4: Micrograph of CM 0 wt% with the spots used for SEM-EDS analysis. BSE mode 3000× magnification.; Table S1: EDS analysis of CM 0 wt%.; Figure S5: Micrograph of CM 0.1 wt% with spots used for SEM-EDS analysis. BSE mode 1500× magnification.; Table S2: EDS analysis of CM 0.1 wt%.; Figure S6: Micrograph of CM 0.2 wt% with spots used for SEM-EDS analysis. BSE mode 1500× magnification.; Table S3: EDS analysis of CM 0.2 wt%.; Figure S7: Micrograph of CM 0.3 wt% with spots used for SEM-EDS analysis. BSE mode 3000× magnification.; Table S4: EDS analysis of CM 0.3 wt%.; Figure S8: Micrograph of CM 0.5 wt% with spots used for SEM-EDS analysis. BSE mode 3000× magnification.; Table S5: EDS analysis of CM 0.5 wt%.; Figure S9: Micrograph of CM 0.8 wt% with spots used for SEM-EDS analysis. BSE mode 3000× magnification.; Table S6: EDS analysis of CM 0.8 wt%.; Figure S10: Micrograph of CM 1 wt% with spots used for SEM-EDS analysis. BSE mode 3000× magnification.; Table S7: EDS analysis of CM 1 wt%.; Figure S11: Micrograph of the MoS_2_ nanopowders with the area used for SE-EDS analysis. BSE mode 22,500× magnification.; Table S8: EDS analysis of the MoS_2_ nanopowders.; Figure S12: Micrographs of CM 0 wt% in SE mode 1000× magnification, (a) pellet center, (b) pellet top, (c) pellet bottom, and (d) example of *ImageJ* mask for porosity calculation.; Figure S13: Micrographs of CM 0.1 wt% in SE mode 1000× magnification, (a) pellet center, (b) pellet top, (c) pellet bottom, and (d) example of *ImageJ* mask for porosity calculation.; Figure S14: Micrographs of CM 0.2 wt% in SE mode 1000× magnification, (a) pellet center, (b) pellet top, (c) pellet bottom, and (d) example of *ImageJ* mask for porosity calculation.; Figure S15: Micrographs of CM 0.3 wt% in SE mode 1000× magnification, (a) pellet center, (b) pellet top, (c) pellet bottom, and (d) example of *ImageJ* mask for porosity calculation.; Figure S16: Micrographs of CM 0.5 wt% in SE mode 1000× magnification, (a) pellet center, (b) pellet top, (c) pellet bottom, and (d) example of *ImageJ* mask for porosity calculation.; Figure S17: Micrographs of CM 0.8 wt% in SE mode 1000× magnification, (a) pellet center, (b) pellet top, (c) pellet bottom, and (d) example of *ImageJ* mask for porosity calculation.; Figure S18: Micrographs of CM 1 wt% in SE mode 1000× magnification, (a) pellet center, (b) pellet top, (c) pellet bottom, and (d) example of *ImageJ* mask for porosity calculation.; Figure S19: Raman Maps for CM 0.2 wt%, (a) map containing the Tetrahedrite matrix signal, (b) map containing the MoS_2_ NPs signal, and (c) Raman basis spectra used to build the maps. Each pixel color in the Raman maps corresponds to the color of the corresponding basis Raman spectra.
