# Supplementary material for: Tetrahedrite Nanocomposites for High Performance Thermoelectrics
Source: Nanomaterials (Basel). 2025 Feb 24;15(5):351. doi: 10.3390/nano15050351 (PMC11902260; doi:10.3390/nano15050351)
Supplement: Supplementary file 1 [file nanomaterials-15-00351-s001.zip › nanomaterials-3471011-supplementary.pdf]

nanomaterials

Supplementary Material

Tetrahedrite Nanocomposites for High Performance Thermoelectrics

Rodrigo Coelho [1], Duarte Moço [1], Ana I. de Sá [2], Paulo P. da Luz [2], Filipe Neves [2], Maria de Fátima Cerqueira [3,4], Elsa B. Lopes [1], Francisco P. Brito [5], Panagiotis Mangelis [6], Theodora Kyratsi [6] and António P. Gonçalves [1,*]

[1] Centro de Ciências e Tecnologias Nucleares (C2TN), Departamento de Engenharia e Ciências Nucleares (DECN), Instituto Superior Técnico, Universidade de Lisboa, Campus Tecnológico e Nuclear, 2695-066 Bobadela, Portugal; rodrigo.coelho@ctn.tecnico.ulisboa.pt (R.C.); duarte_moco@sapo.pt (D.M.); eblopes@ctn.tecnico.ulisboa.pt (E.B.L.)
[2] Laboratório Nacional de Energia e Geologia, I.P., Campus do Lumiar, Estrada do Paço do Lumiar, 22, 1649-038 Lisboa, Portugal; ana.sa@lneg.pt (A.I.d.S.); paulo.luz@lneg.pt (P.P.d.L.); filipe.neves@lneg.pt (F.N.)
[3] International Iberian Nanotechnology Laboratory, 4715-330 Braga, Portugal; fatima.cerqueira@inl.int
[4] Centro de Física das Universidades do Minho e Porto (CF-UM-UP), Universidade do Minho, 4710-057 Braga, Portugal
[5] Mechanical Engineering and Resource Sustainability Center (MEtRICs), Departamento de Engenharia Mecânica (DEM), Universidade do Minho, 4800-058 Guimarães, Portugal; francisco@dem.uminho.pt
[6] Department of Mechanical and Manufacturing Engineering, University of Cyprus, 1678 Nicosia, Cyprus; mangelis.panagiotis@ucy.ac.cy (P.M.); kyratsi.theodora@ucy.ac.cy (T.K.)
* Correspondence: apg@ctn.tecnico.ulisboa.pt; Tel.: +351-219946182

Citation: To be added by editorial staff during production.

Academic Editor: Firstname Lastname

Received: date
Revised: date
Accepted: date
Published: date

Copyright: © 2024 by the authors. Submitted for possible open access publication under the terms and conditions of the Creative Commons Attribution (CC BY) license (https://creativecommons.org/licenses/by/4.0/).

Presented in Figure S1 is displayed the MoS_2 nanopartciles (NPs) XRD analysis. Along with the NPs diffractogram there is presented the pattern simulation using the COD card # 9007660.

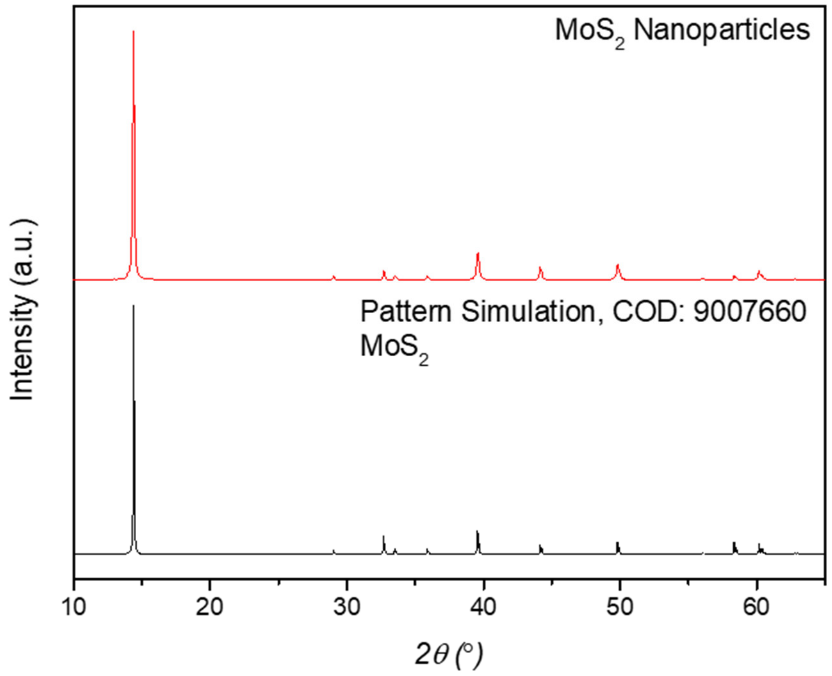

Figure S1. XRD analysis of the MoS_2 NPs on the top, and pattern simulation using COD card #9007660 on the bottom.

The cell parameters, average crystallite size, and microstrain in function of MoS_2 NPs addition for all the manganese composites are presented in Figure S2. The cell parameter "*a*" was calculated using the *UnitCell* program. To account for instrumental errors like sample displacement and misalignment (during XRD analysis), the refine zero-shift option was enabled. The average Crystallite size and microstrain were obtained by generating the Williamson-Hall (W-H) plots that are present in Figure S3. The equation presented in each graph displays the slope and intercept (Kλ/D), which according to the W-H method corresponds to the microstrain and the average crystallite size, respectively. The W-H plots are based on the Uniform Deformation Model (UDM). Each data point was obtained from the XRD analysis of the pellets by performing a Gaussian fit (Adj. R-Square of ~0.95) followed by the application of UDM. In the graphs, the squares represent the crystalline peaks corresponding to the (*hkl*) planes. The x-axis is labelled as ($4\ sin\theta$) and the y-axis as ($\beta\ cos\theta$), where θ is the diffraction angle and β the full width at half maximum (FWHM) of the peaks.

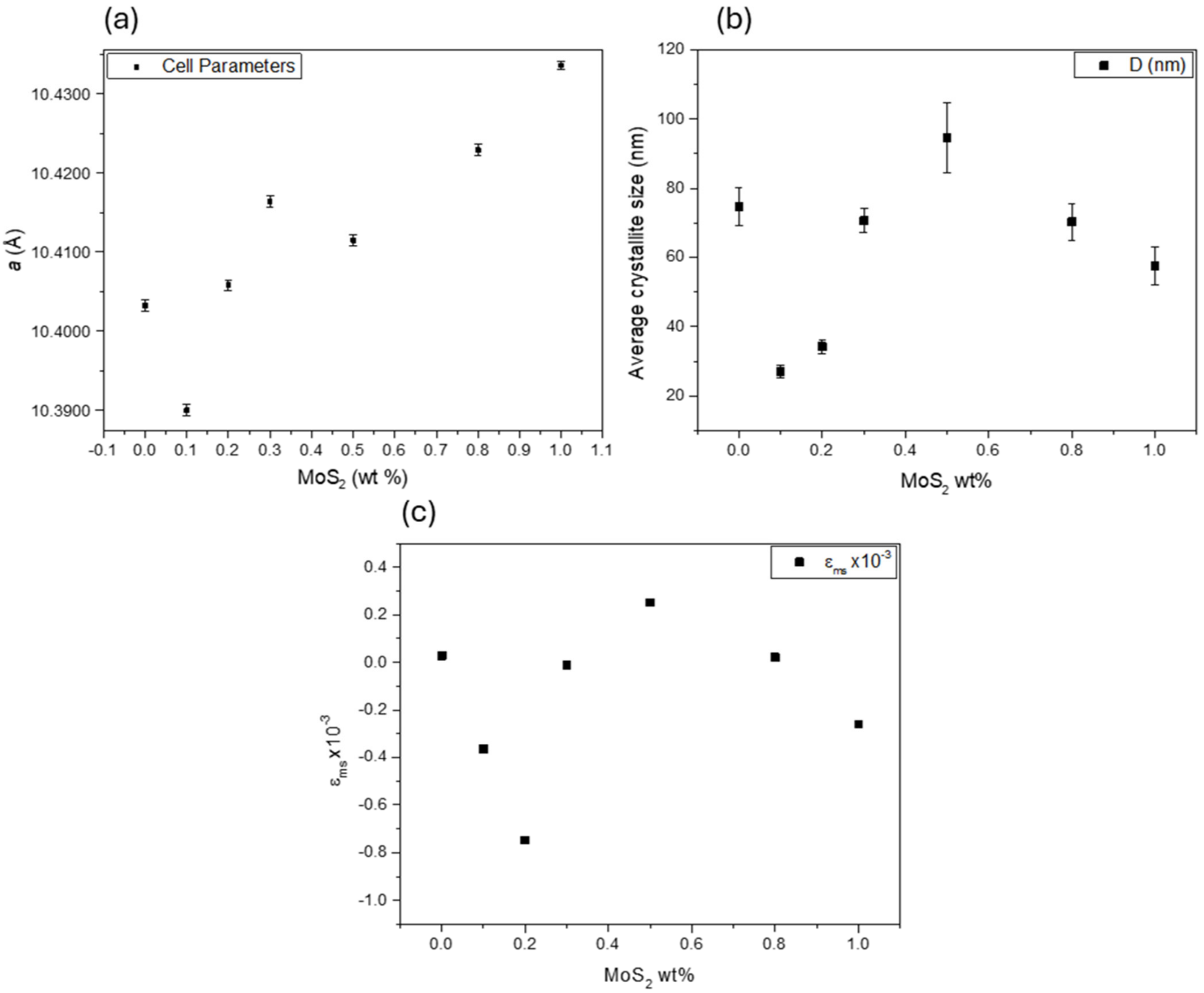

Figure S2. Tetrahedrite composites cell parameters a), average crystallite size b), and lattice strain c).

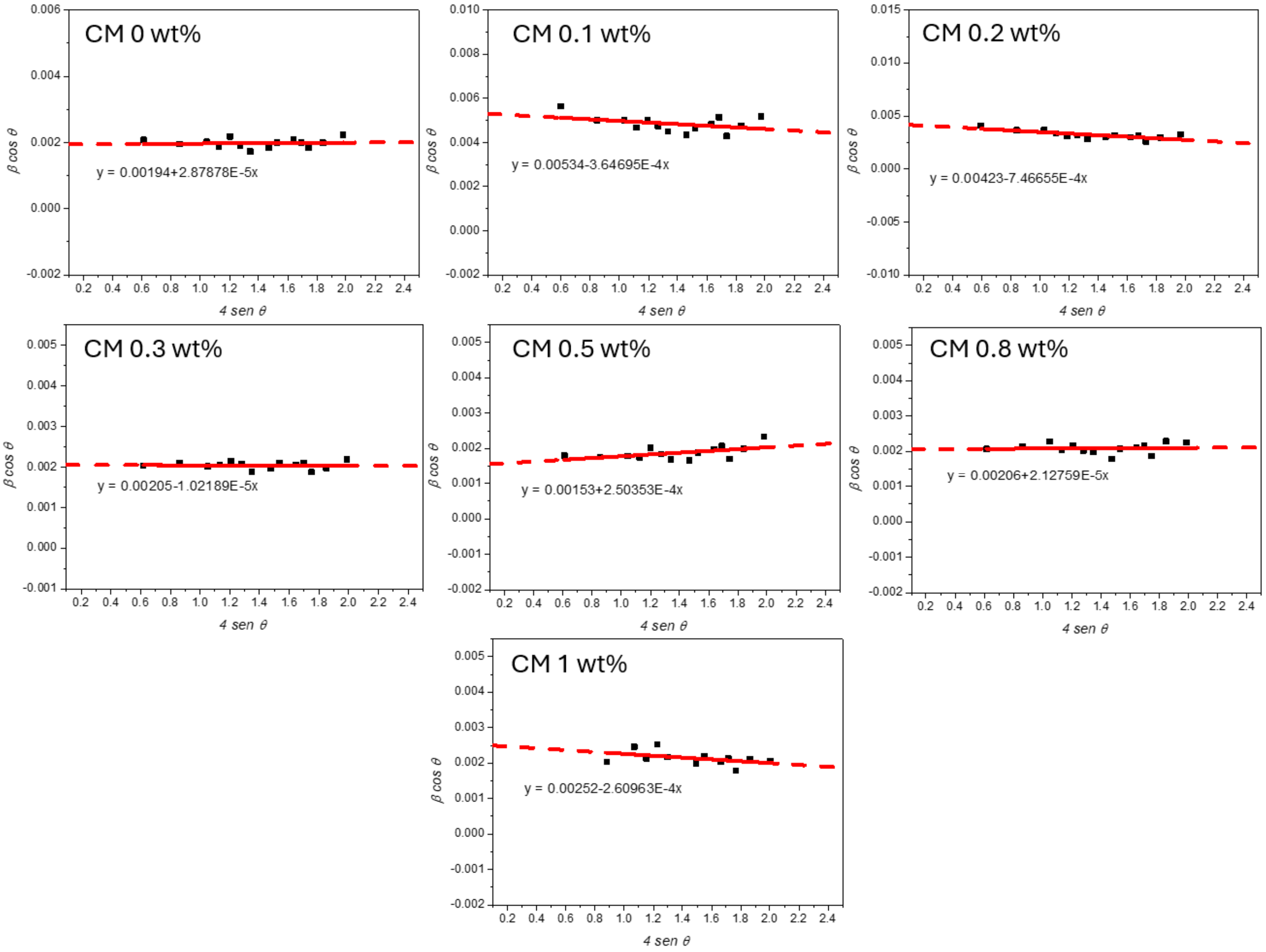

Figure S3. W-H plots of the tetrahedrite composites employing the UDM.

The detailed SEM-EDS analysis of the composite materials can be observed from Figure S4 up to Figure S11. The spots where the EDS analysis was preformed are marked by arrows and numbers. Below each micrograph, there are presented the tables containing the summary of the semi-quantitative EDS analysis with the chemical composition of the indicated zones displayed in at%.

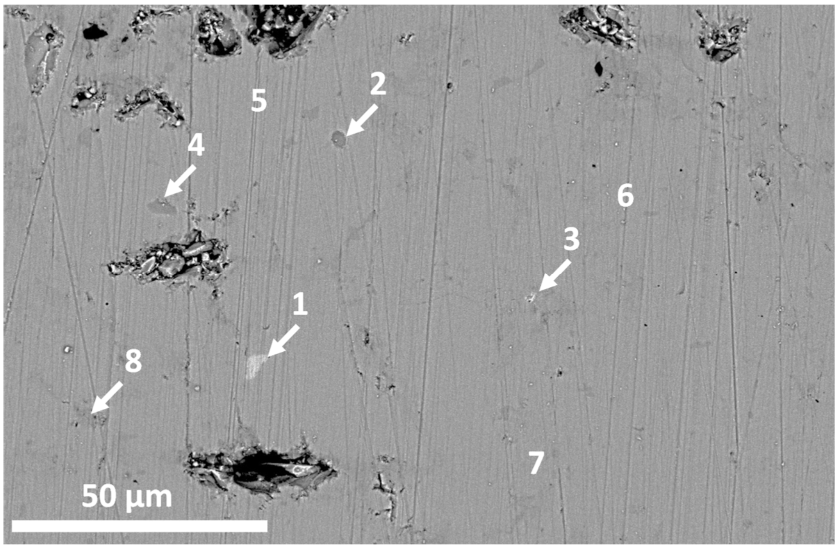

Figure S4. Micrograph of CM 0 wt % with the spots used for the SEM-EDS analysis. BSE mode 3000 x magnification.

Table S1. EDS analysis of CM 0 wt %.

		Composition at %				
**Zone**	**Phase type**	**Cu**	**Mn**	**Sb**	**S**	**O**
**1**	**Cu_3SbS_4**	28 (3)	-	19 (2)	53 (6)	-
**2**	**$Cu_{12}Sb_4S_{13}$**	37 (4)	4 (1)	12 (1)	47 (6)	-
**3**	**SbO_2 and $Cu_{12}Sb_4S_{13}$**	34 (4)	-	20 (2)	21 (3)	25 (3)
**4**	**$Cu_{12}Sb_4S_{13}$**	39 (5)	3 (1)	11 (1)	47 (6)	-
**5**	**$Cu_{12}Sb_4S_{13}$**	41 (5)	3 (1)	10 (1)	46 (6)	-
**6**	**$Cu_{12}Sb_4S_{13}$**	41 (5)	3 (1)	10 (1)	46 (6)	-
**7**	**$Cu_{12}Sb_4S_{13}$**	41 (5)	3 (1)	10 (1)	46 (6)	-
**8**	**$Cu_{12}Sb_4S_{13}$**	43 (5)	2 (1)	8 (1)	40 (5)	7 (1)

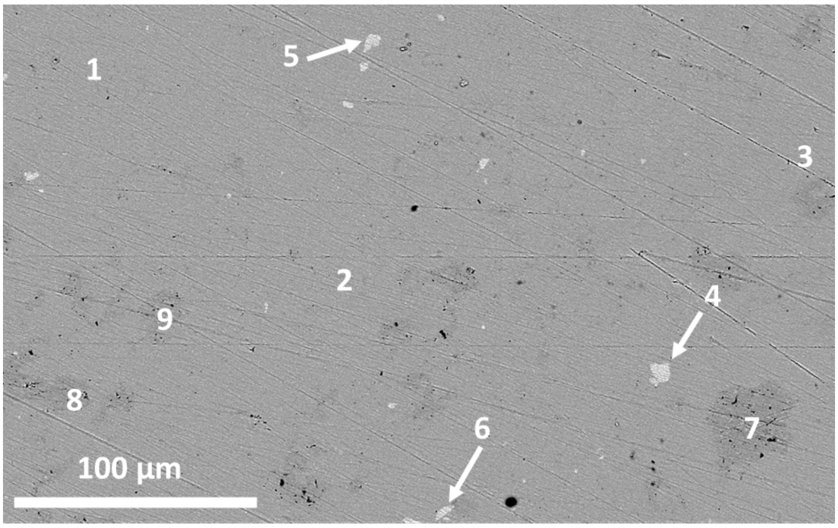

Figure S5. Micrograph of CM 0.1 wt % with the spots used for the SEM-EDS analysis. BSE mode 1500 x magnification.

Table S2. EDS analysis of CM 0.1 wt %.

		**Composition at %**				
**Zone**	**Phase type**	**Cu**	**Mn**	**Sb**	**S**	**O**
**1**	$\mathbf{Cu_{12}Sb_4S_{13}}$	36 (4)	4 (1)	10 (1)	50 (6)	-
**2**	$\mathbf{Cu_{12}Sb_4S_{13}}$	37 (4)	3 (1)	10 (1)	50 (6)	-
**3**	$\mathbf{Cu_{12}Sb_4S_{13}}$	35 (4)	4 (1)	10 (1)	51 (6)	-
**4**	$\mathbf{CuSbS_2}$	26 (3)	-	18 (2)	56 (7)	-
**5**	$\mathbf{CuSbS_2}$	26 (3)	-	18 (2)	56 (7)	-
**6**	$\mathbf{CuSbS_2}$	27 (3)	-	18 (2)	55 (7)	-
**7**	$\mathbf{Cu_{12}Sb_4S_{13}}$	36 (4)	2 (1)	9 (1)	45 (5)	8 (1)
**8**	$\mathbf{Cu_{12}Sb_4S_{13}}$	37 (4)	2 (1)	9 (1)	43 (5)	9 (1)
**9**	$\mathbf{Cu_{12}Sb_4S_{13}}$	38 (5)	3 (1)	9 (1)	42 (5)	8 (1)

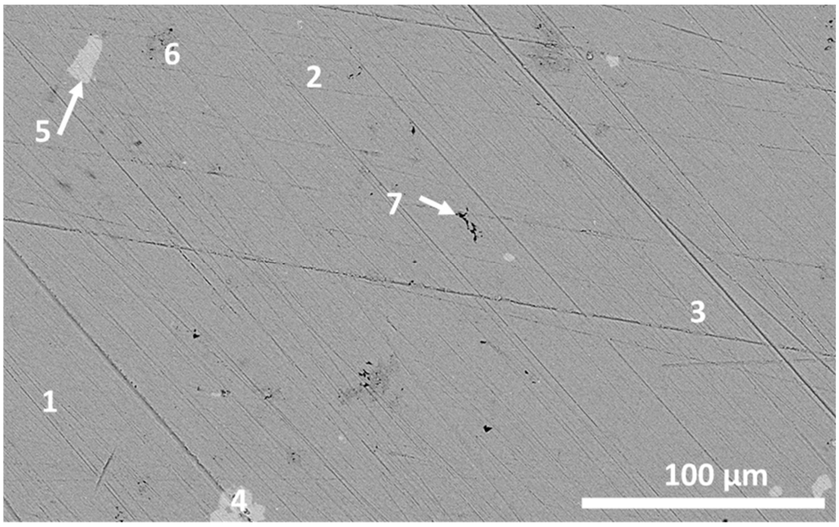

Figure S6. Micrograph of CM 0.2 wt % with the spots used for the SEM-EDS analysis. BSE mode 1500 x magnification.

Table S3. EDS analysis of CM 0.2 wt %.

		**Composition at %**				
**Zone**	**Phase type**	**Cu**	**Mn**	**Sb**	**S**	**O**
**1**	$Cu_{12}Sb_4S_{13}$	38 (5)	3 (1)	10 (1)	49 (6)	-
**2**	$Cu_{12}Sb_4S_{13}$	37 (4)	3 (1)	10 (1)	50 (6)	-
**3**	$Cu_{12}Sb_4S_{13}$	36 (4)	4 (1)	10 (1)	50 (6)	-
**4**	$CuSbS_2$	26 (3)	-	18 (2)	56 (7)	-
**5**	$CuSbS_2$	27 (3)	-	17 (2)	56 (7)	-
**6**	$Cu_{12}Sb_4S_{13}$	38 (5)	2 (1)	8 (1)	43 (5)	9 (1)
**7**	$MnSO_4$	7 (1)	11 (1)	2 (1)	21 (3)	59 (7)

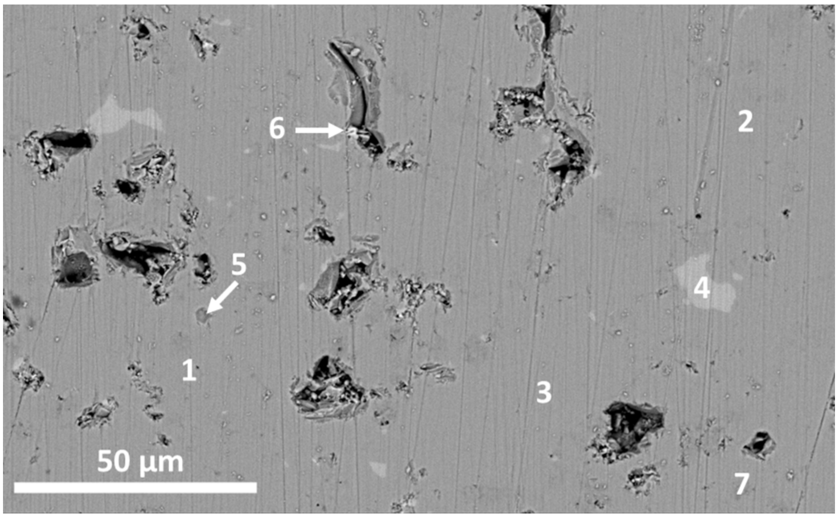

Figure S7. Micrograph of CM 0.3 wt % with the spots used for the SEM-EDS analysis. BSE mode 3000 x magnification.

Table S4. EDS analysis of CM 0.3 wt %.

		Composition at %				
**Zone**	**Phase type**	**Cu**	**Mn**	**Sb**	**S**	**O**
**1**	**$Cu_{12}Sb_4S_{13}$**	40 (5)	4 (1)	10 (1)	46 (6)	-
**2**	**$Cu_{12}Sb_4S_{13}$**	37 (4)	3 (1)	8 (1)	52 (6)	-
**3**	**$Cu_{12}Sb_4S_{13}$**	40 (5)	3 (1)	10 (1)	47 (6)	-
**4**	**$CuSbS_2$**	28 (3)	-	19 (2)	53 (6)	-
**5**	**$Cu_{12}Sb_4S_{13}$**	44 (5)	3 (1)	9 (1)	44 (5)	-
**6**	**SbO_2 and $Cu_{12}Sb_4S_{13}$**	13 (2)	1 (1)	35 (4)	8 (1)	43 (5)
**7**	**$Cu_{12}Sb_4S_{13}$**	45 (5)	2 (1)	7 (1)	36 (4)	10 (4)

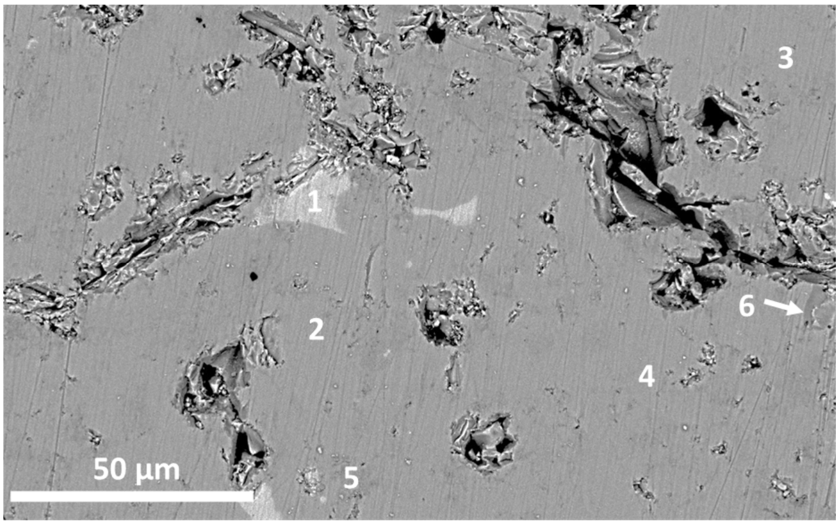

Figure S8. Micrograph of CM 0.5 wt % with the spots used for the SEM-EDS analysis. BSE mode 3000 x magnification.

Table S5. EDS analysis of CM 0.5 wt %.

		Composition at %				
**Zone**	**Phase type**	**Cu**	**Mn**	**Sb**	**S**	**O**
**1**	$\mathbf{CuSbS_2}$	28 (3)	-	19 (2)	53 (6)	-
**2**	$\mathbf{Cu_{12}Sb_4S_{13}}$	40 (5)	4 (1)	10 (1)	46 (6)	-
**3**	$\mathbf{Cu_{12}Sb_4S_{13}}$	41 (5)	3 (1)	10 (1)	46 (6)	-
**4**	$\mathbf{Cu_{12}Sb_4S_{13}}$	41 (5)	4 (1)	10 (1)	45 (5)	-
**5**	$\mathbf{Cu_{12}Sb_4S_{13}}$	42 (5)	3 (1)	10 (1)	45 (5)	-
**6**	$\mathbf{Cu_{12}Sb_4S_{13}}$	37 (4)	4 (1)	11 (1)	48 (6)	-

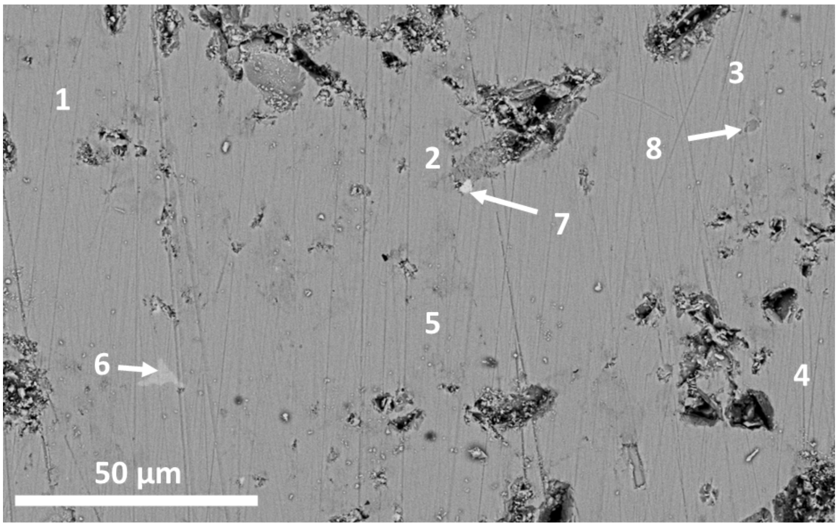

Figure S9. Micrograph of CM 0.8 wt % with the spots used for the SEM-EDS analysis. BSE mode 3000 x magnification.

Table S6. EDS analysis of CM 0.8 wt %.

		**Composition at %**				
**Zone**	**Phase type**	**Cu**	**Mn**	**Sb**	**S**	**O**
**1**	$\mathbf{Cu_{12}Sb_4S_{13}}$	41 (5)	3 (1)	10 (1)	46 (6)	-
**2**	$\mathbf{Cu_{12}Sb_4S_{13}}$	41 (5)	3 (1)	10 (1)	46 (6)	-
**3**	$\mathbf{Cu_{12}Sb_4S_{13}}$	41 (5)	3 (1)	10 (1)	46 (6)	-
**4**	$\mathbf{Cu_{12}Sb_4S_{13}}$	41 (5)	3 (1)	10 (1)	46 (6)	-
**5**	$\mathbf{Cu_{12}Sb_4S_{13}}$	41 (5)	3 (1)	10 (1)	46 (6)	-
**6**	$\mathbf{CuSbS_2}$	30 (4)	-	18 (2)	52 (6)	-
**7**	$\mathbf{SbO_2}$	4 (1)	-	31 (4)	5 (1)	60 (7)
**8**	$\mathbf{Cu_{12}Sb_4S_{13}}$	37 (4)	4 (1)	11 (1)	48 (6)	-

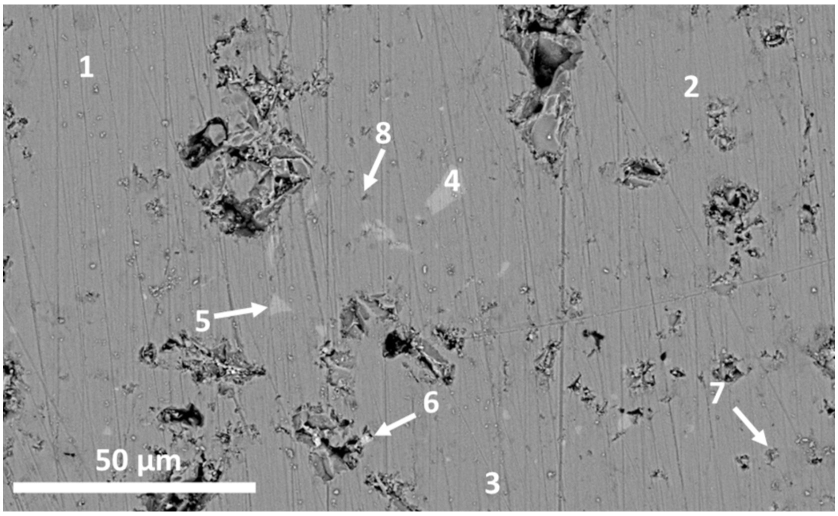

Figure S10. Micrograph of CM 1 wt % with the spots used for the SEM-EDS analysis. BSE mode 3000 x magnification.

Table S7. EDS analysis of CM 1 wt %.

		**Composition at %**					
**Zone**	**Phase type**	**Cu**	**Mn**	**Sb**	**S**	**O**	**Mo**
**1**	**$Cu_{12}Sb_4S_{13}$**	40 (5)	4 (1)	10 (1)	46 (6)	-	-
**2**	**$Cu_{12}Sb_4S_{13}$**	40 (5)	4 (1)	10 (1)	46 (6)	-	-
**3**	**$Cu_{12}Sb_4S_{13}$**	39 (5)	3 (1)	10 (1)	48 (6)	-	-
**4**	**$CuSbS_2$**	29 (4)	-	18 (2)	53 (6)	-	-
**5**	**$CuSbS_2$**	29 (4)	-	18 (2)	53 (6)	-	-
**6**	**SbO_2 and $Cu_{12}Sb_4S_{13}$**	11 (1)	-	26 (3)	7 (1)	56 (7)	-
**7**	**$Cu_{12}Sb_4S_{13}$**	35 (4)	3 (1)	9 (1)	53 (6)	-	-
**8**	**MoS_2 and $Cu_{12}Sb_4S_{13}$**	34 (4)	2 (1)	8 (1)	48 (6)	-	8 (1)

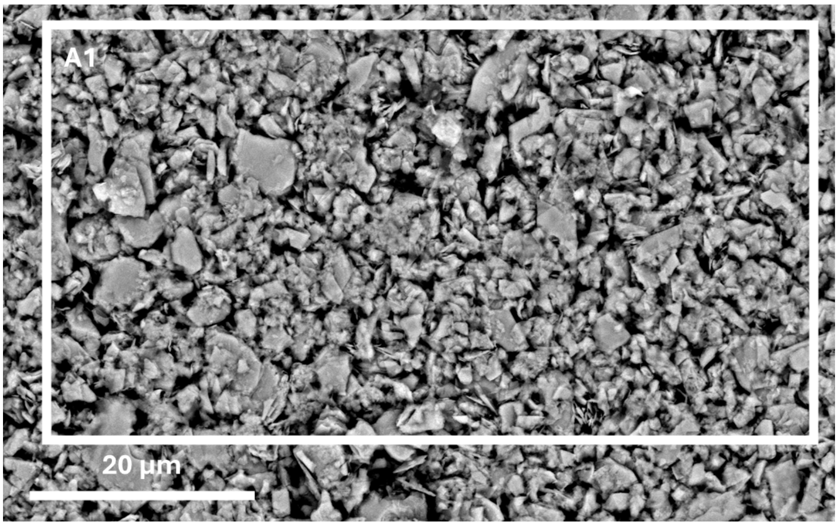

Figure S11. Micrograph of the MoS_2 nano-powder with the area used for SE-EDS analysis. BSE mode 22500 x magnification.

Table S8. EDS analysis of the MoS_2 nanopowders.

		**Composition at %**	
**Zone**	**Phase type**	**Mo**	**S**
**A1**	**MoS_2**	36 (4)	64 (8)

The SEM micrographs acquired for the porosity analysis are presented from Figure S12 up to Figure S18. On the SEM micrographs, the bright and drack regions correspond to porous and debris that result from the polishing process. The porosity was estimated using the *ImageJ* program by adjusting the images threshold followed by the application of a particle analysis method. The zones considered as pores are displayed in black in the micrograph masks.

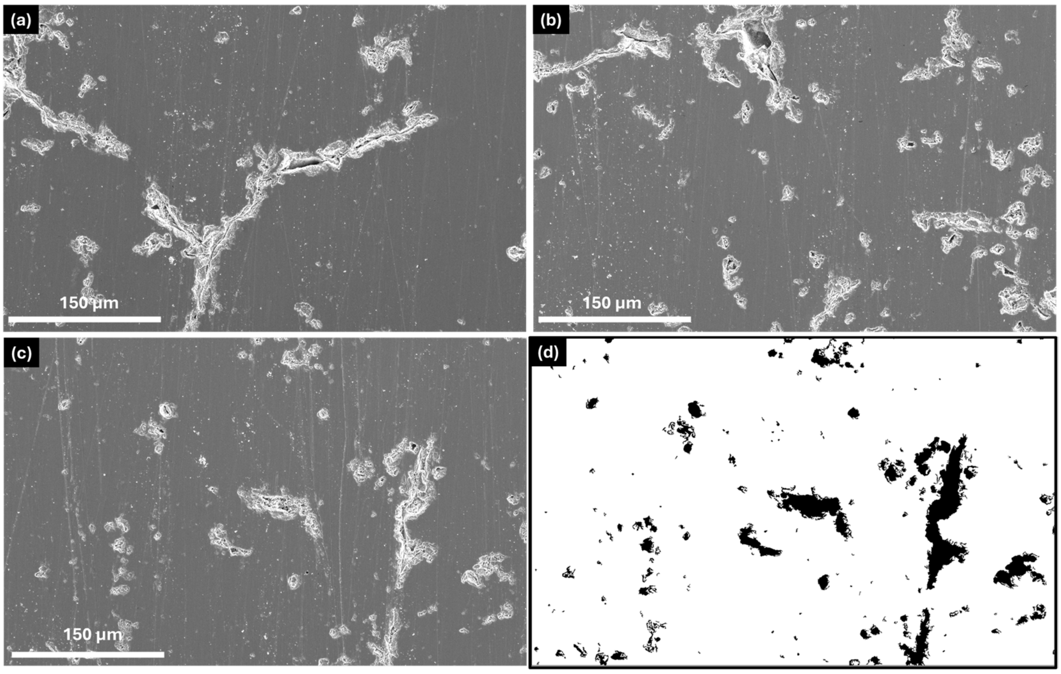

Figure S12. Micrographs of CM 0 wt% in SE mode 1000 x magnification, a) pellet centre, b) pellet top, c) pellet bottom, and d) example of *ImageJ* mask for porosity calculation.

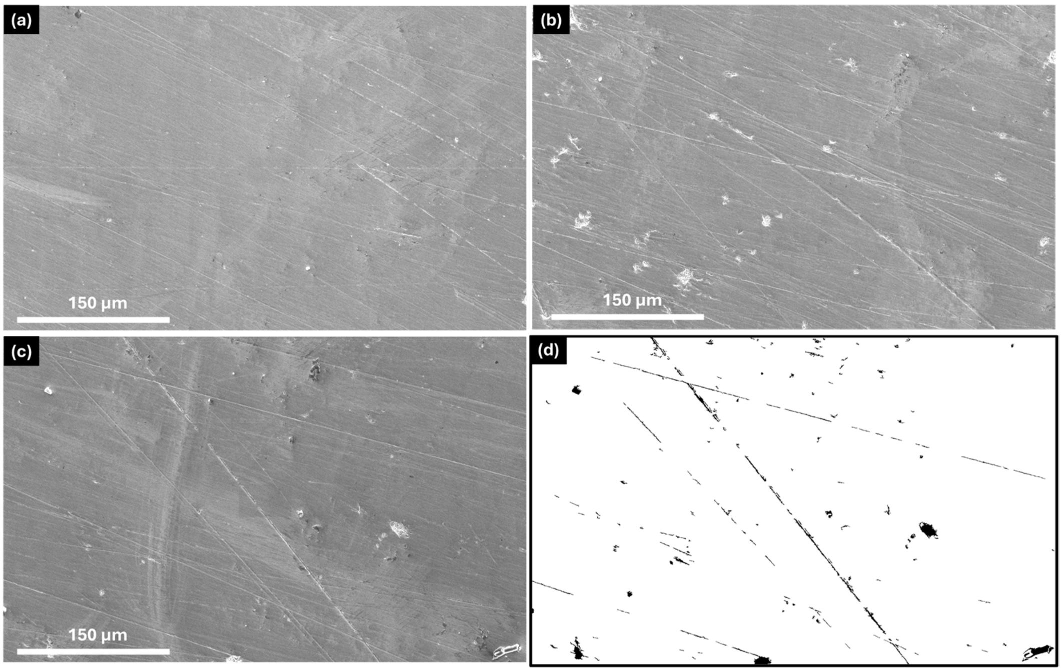

Figure S13. Micrographs of CM 0.1 wt% in SE mode 1000 x magnification, a) pellet centre, b) pellet top, c) pellet bottom, and d) example of *ImageJ* mask for porosity calculation.

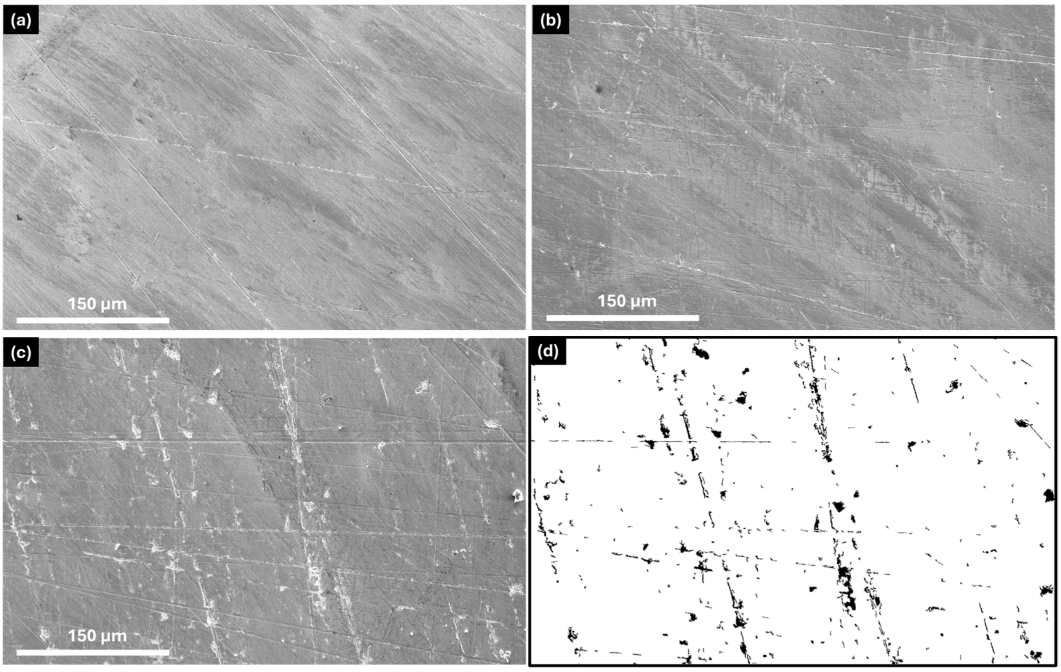

Figure S14. Micrographs of CM 0.2 wt% in SE mode 1000 x magnification, a) pellet centre, b) pellet top, c) pellet bottom, and d) example of *ImageJ* mask for porosity calculation.

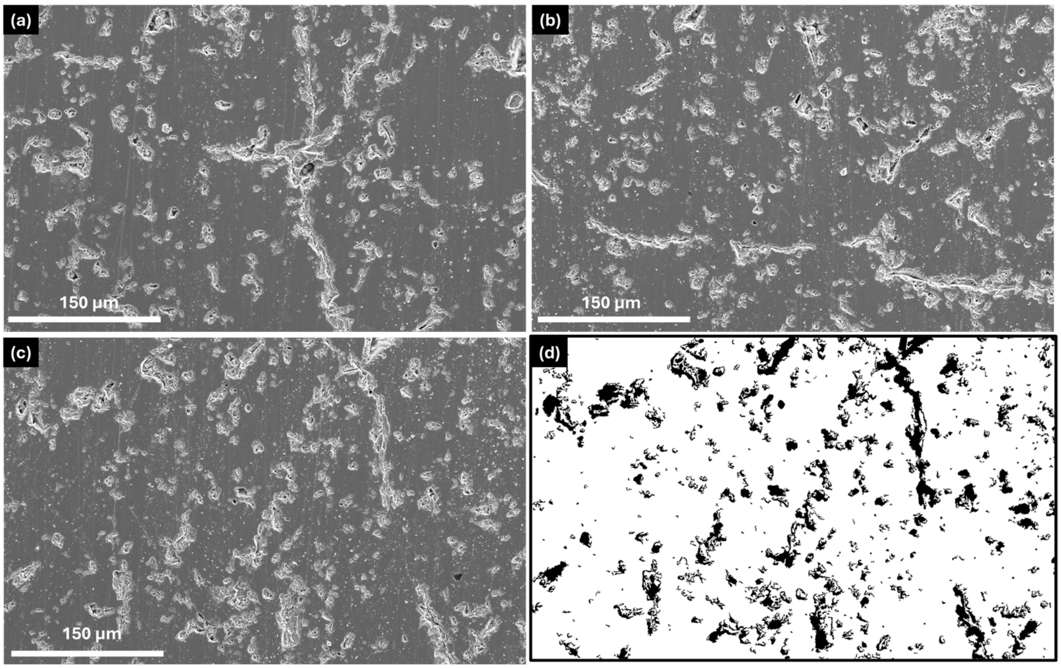

Figure S15. Micrographs of CM 0.3 wt% in SE mode 1000 x magnification, a) pellet centre, b) pellet top, c) pellet bottom, and d) example of *ImageJ* mask for porosity calculation.

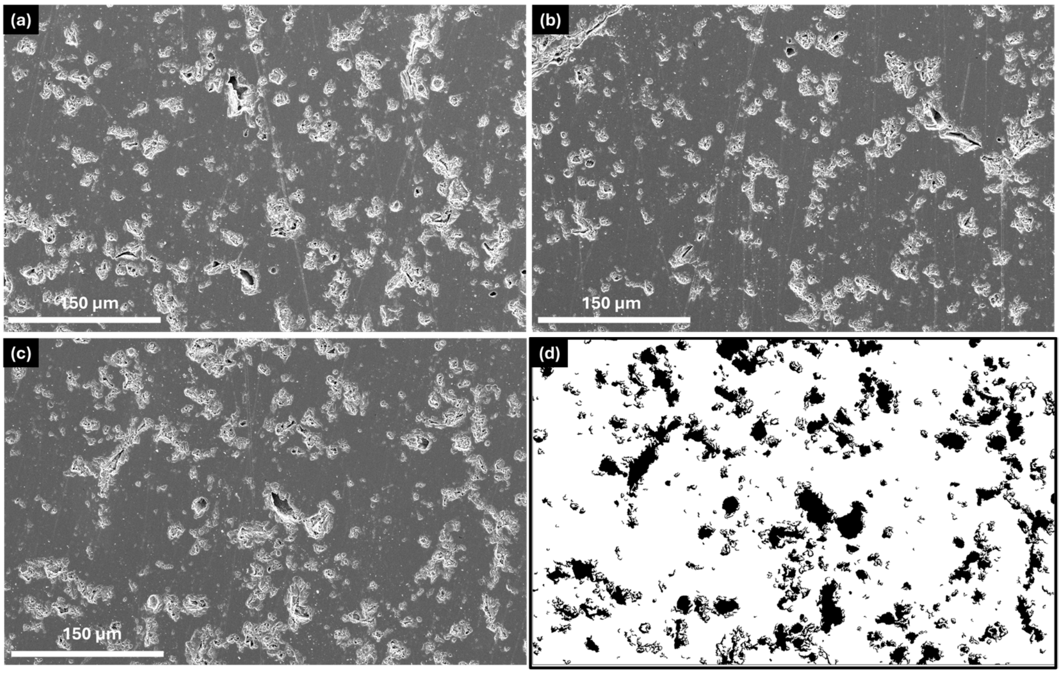

Figure S16. Micrographs of CM 0.5 wt% in SE mode 1000 x magnification, a) pellet centre, b) pellet top, c) pellet bottom, and d) example of *ImageJ* mask for porosity calculation.

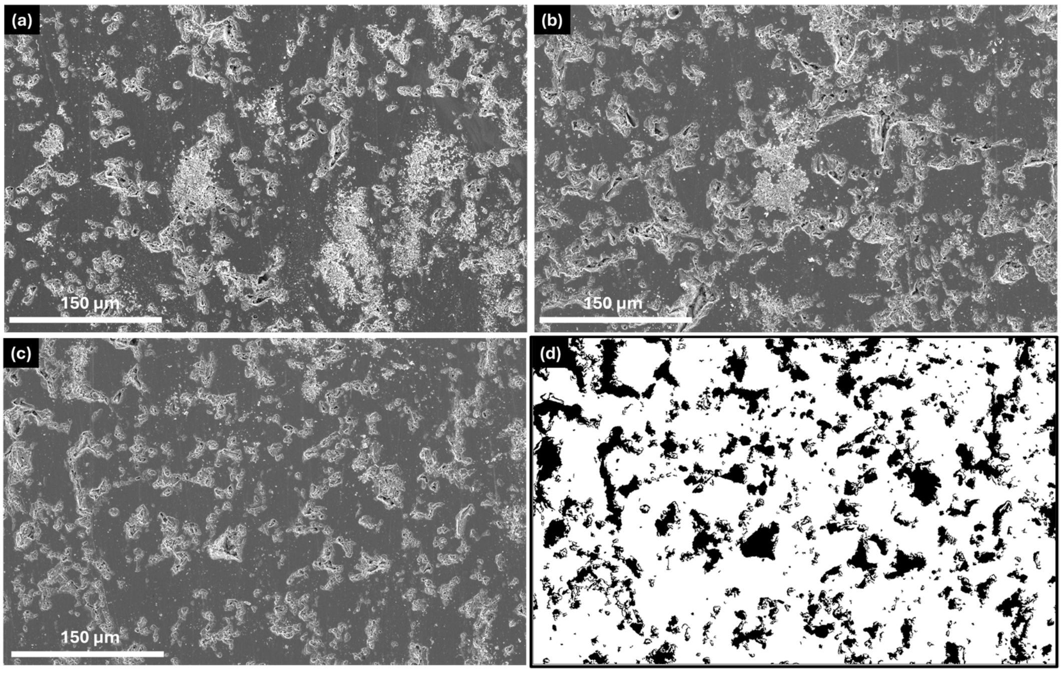

Figure S17. Micrographs of CM 0.8 wt% in SE mode 1000 x magnification, a) pellet centre, b) pellet top, c) pellet bottom, and d) example of *ImageJ* mask for porosity calculation.

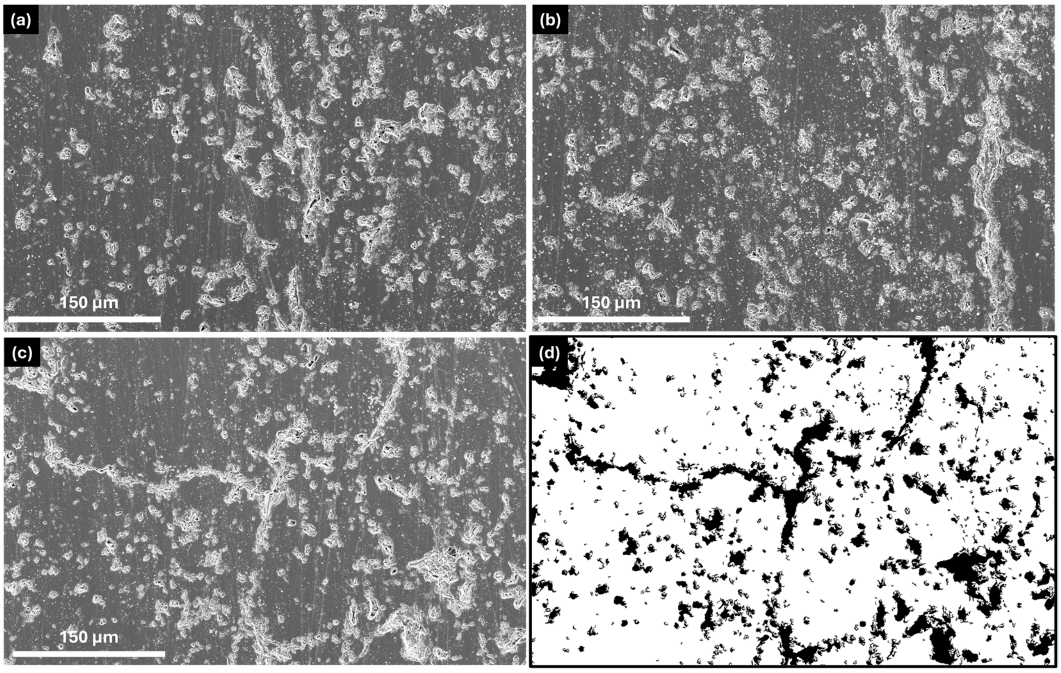

Figure S18. Micrographs of CM 1 wt% in SE mode 1000 x magnification, a) pellet centre, b) pellet top, c) pellet bottom, and d) example of *ImageJ* mask for porosity calculation.

The Raman maps acquired from the surface of the composite pellet filled with 0.2 wt% of MoS_2 are depicted in Figure S19.The maps at each side of the figure contain the isolated contribution of only 1 signal (1 data cluster). The map on the right, Figure S19 -a) contains the signal of the MoS_2 NPs, while the map on the left, Figure S19 -b), contains only the signal of the tetrahedrite matrix. On the bottom, Figure S19 -c), there is displayed the respective Raman spectra used for the generation of the maps.

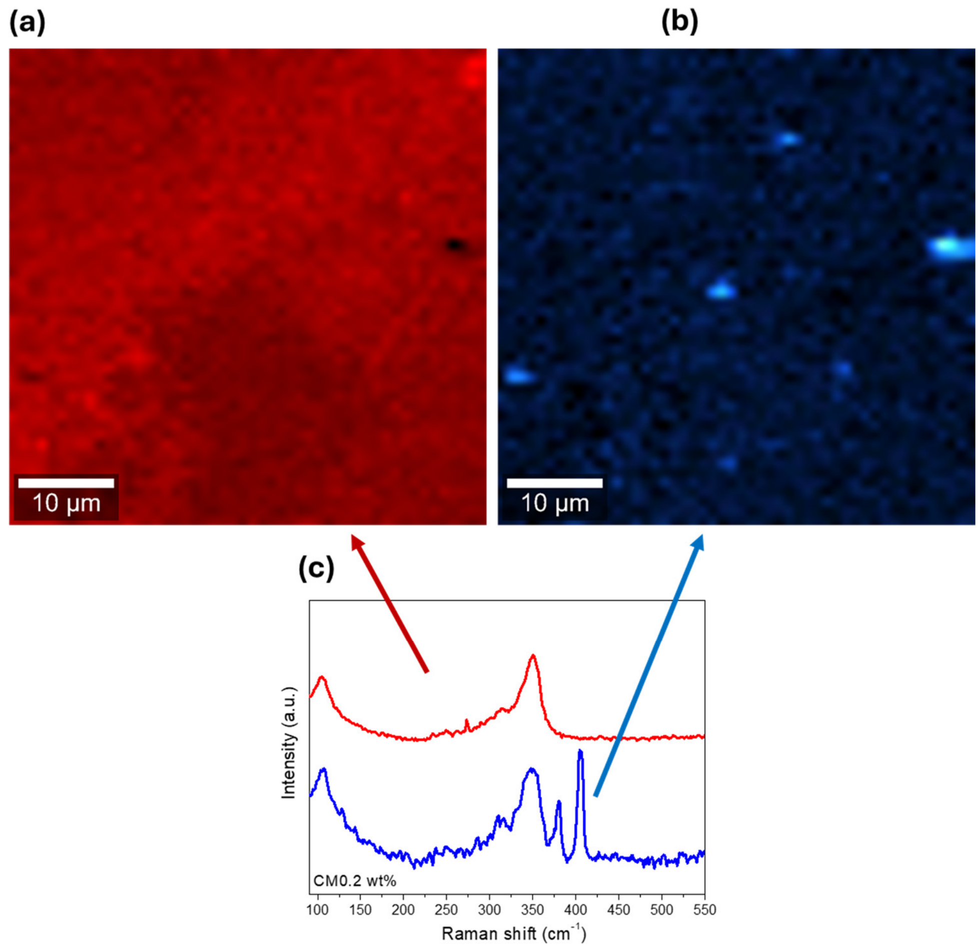

Figure S19. Raman Maps for CM 0.2 wt%, a) map containing the Tetrahedrite matrix signal, b) map containing the MoS_2 NPs signal, c) Raman Basis spectra used to build the maps. Each pixel color in the Raman maps corresponds to the color of the corresponding basis Raman spectra.
